# Lipid-lowering drugs and essential hemorrhagic thrombocythemia’s risk: A drug-target Mendelian randomization study

**DOI:** 10.1097/MD.0000000000049077

**Published:** 2026-05-29

**Authors:** Peng Zhang, HaiJiao Wang, MingHao Lin, Peng Dai, Qi Zhong, RuoLin Wang, Lei Sheng, Ze He, Zheng Nan

**Affiliations:** a Changchun University of Traditional Chinese Medicine, Changchun, China; b Affiliated Hospital of Changchun University of Chinese Medicine, Changchun, China.

**Keywords:** drug-target, essential hemorrhagic thrombocythemia, lipid-lowering drugs, Mendelian randomization

## Abstract

Lipid-lowering drugs are currently widely used by clinicians in clinical practice; numerous related studies have confirmed the pivotal role of the lipid pathway in regulating platelet function. Lipid-lowering drugs, represented by statins, have been found to have an improving effect on platelet-related diseases. However, there is a lack of large-scale population studies at present, and the causal explanation of traditional observational design studies is easily limited by confounding variables. The impact of lipid-lowering drugs on the risk of essential hemorrhagic thrombocythemia is not clearly understood. Our study sought to investigate the causal association between essential hemorrhagic thrombocythemia and lipid-lowering drugs through a drug-target Mendelian randomization (MR) analysis. We utilized the data from the Global Lipid Genetics Consortium to identify instrumental variables for 3 types of lipid-lowering drugs (3-hydroxy-3-methylglutaryl-CoA reductase inhibitors, proprotein convertase subtilisin/kexin type 9 [PCSK9] inhibitors, and NPC1-like intracellular cholesterol transporter 1 [NPC1L1] inhibitors). We obtained the genome-wide association study data for essential hemorrhagic thrombocythemia from the FinnGen study. We employed the MR method based on aggregated data and the inverse variance weighted method for the analysis. Sensitivity analyses were conducted using the conventional MR method. By analyzing data from 1171 patients with essential hemorrhagic thrombocythemia and about 1.3 million individuals with low-density lipoprotein (LDL) testing, PCSK9 inhibition was associated with a significantly increased risk of essential hemorrhagic thrombocythemia (odds ratio [OR], LDL ratio of 1.87 for every 1 standard deviation increased; 95% confidence interval [CI] = 1.2–2.91; *P* = .02). NPC1L1 inhibition was associated with a reduced risk of essential hemorrhagic thrombocythemia (OR, LDL 0.19 for every 1 standard deviation reduction; 95% CI = 0.05–0.73; *P* = .03). No association was found between 3-hydroxy-3-methylglutaryl-CoA reductase inhibition and essential hemorrhagic thrombocythemia (OR = 0.76, 95% CI = 0.34–1.69, *P* = .51). The results of this MR study suggest that NPC1L1 inhibition is causally associated with a reduction in primary hemorrhagic thrombocythemia, whereas PCSK9 inhibition was positively correlated with the occurrence of the disease, which provided a new clue for the future treatment of essential hemorrhagic thrombocythemia.

## 1. Introduction

“Essential hemorrhagic thrombocythemia” specifically refers to essential thrombocythemia (ET), as defined by the World Health Organization Classification of Myeloproliferative Neoplasms. “Hemorrhagic” refers to the phenotype of ET hemorrhagic complications that are caused by a qualitative platelet defect (e.g., acquired von Willebrand syndrome) rather than a distinct clinical subtype. Essential hemorrhagic thrombocythemia is often asymptomatic at clinical diagnosis, or only presents with microvascular symptoms such as erythromelalgia, headache, or may be accompanied by mild splenomegaly (<5 cm). The age of onset is mostly between 60 and 70 years old. It can be seen in some women of childbearing age, and it is rare in children.^[1]^ There are only 1.5 to 2 cases per 100,000 people.^[2,3]^ The development of the disease is mainly associated with genetic mutations, and treatment options are limited. The current treatment strategy is to reduce the incidence of vascular events by cytoreduction in high-risk patients.^[4]^

Cholesterol and other fats have been shown to have an important impact on platelet function. Patients with lipid metabolism disorders are often prone to platelet-related problems. It is easy to induce cardiovascular and cerebrovascular diseases and even death. Studies have shown that patients with high levels of cholesterol may have increased platelet aggregation,^[5]^ proposing that specific lipid-lowering medications could potentially offer protection against essential hemorrhagic thrombocythemia. Lowering lipid levels is essential in reducing the risk of cardiovascular disease. Certain lipid-lowering drugs, for example, NPC1-like intracellular cholesterol transporter 1 (NPC1L1) and PCSK9 inhibitors, are thought to improve platelet activity and therefore have potential disease-modifying properties for essential hemorrhagic thrombocythemia.^[6]^

However, the above evidence is observational and may be subject to confounding factors and reverse causation, making it difficult to produce causal evidence. Our article provides a comprehensive and systematic approach to studying the relationship between Mendelian randomization (MR) of drug targets and disease,^[7]^ reducing confounding factors. There are tens of thousands of drug target proteins in the human genome that encode natural variations in genes–drug targets,^[8]^ which can provide evidence for potential drug targets. Through this method, we studied the relationship between lipid-lowering drugs and primary hemorrhagic thrombocytopenia.

## 2. Materials and methods

### 2.1. Materials

**Figure 1.**
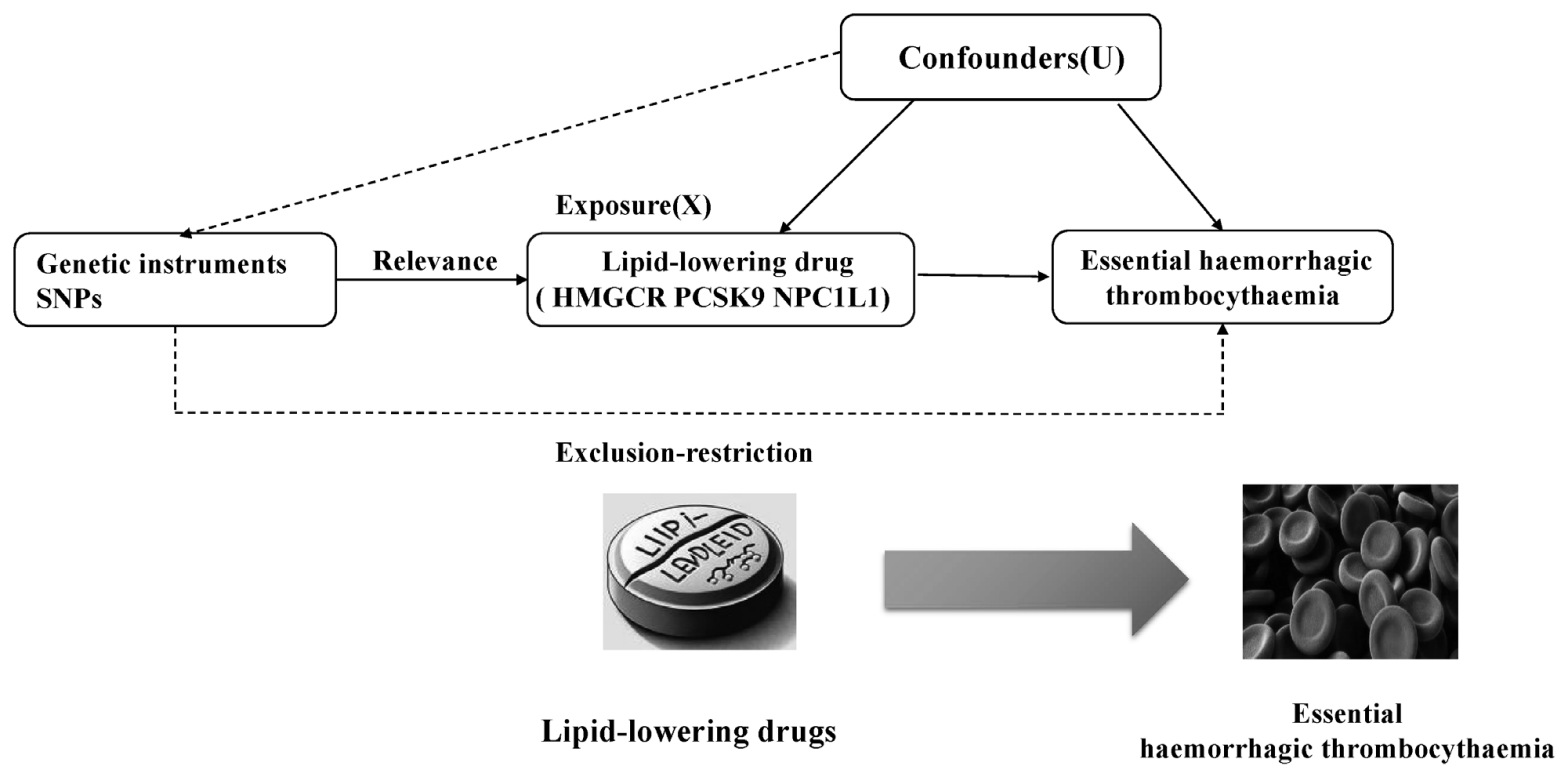
Flow diagram. HMGCR = 3-hydroxy-3-methylglutaryl-CoA reductase, NPC1L1 = NPC1-like intracellular cholesterol transporter 1, PCSK9 = proprotein convertase subtilisin/kexin type 9, SNP = single nucleotide polymorphism.

A genome-wide association study (GWAS) pooled publicly available data were used in the study, which received ethical approval in the original research. The study adhered to the STROBE-MR checklist provided as Supplementary Material (Supplemental Digital Content, Guidelines checklist). An overview of the study design is depicted in Figure 1.

#### 2.1.1. Genetic proxies for lipid-lowering drugs

We chose low-density lipoprotein (LDL) as a biomarker due to the efficacy of lipid-lowering drugs in reducing LDL cholesterol levels. To our knowledge, the genes associated with LDL were identified from the largest GWAS meta-analysis to date. The study, which included about 1.3 million people of European descent, was conducted by the Global Society for Lipid Genetics.^[9,10]^ To mitigate potential bias from sample overlap, analyses utilizing outcomes from FinnGen were conducted using data that excluded Finnish participants (n = 345,506; Table 1).

**Table 1:** Data source.

Dataset	Sample size	Ethnicity	Consortium	Download site
Low-density lipoprotein	1.3 million	European	GLGC	https://csg.sph.umich.edu/willer/public/glgc-lipids2021/
Essential hemorrhagic thrombocythemia	345,506 (1171 cases/344,335 controls)	European	FinnGen	https://www.finngen.fi/en
Coronary artery disease	187,599 (61,289 cases/126,310 controls)	Mixed population	GWAS Catalog	https://www.ebi.ac.uk/gwas/

GLGC = Global Lipids Genetics Consortium, GWAS = genome-wide association study.

#### 2.1.2. Genetic association of essential hemorrhagic thrombocythemia

We obtained GWAS data for essential hemorrhagic thrombocythemia from the FinnGen study (https://www.finngen.fi/en). It is considered to have primary myelofibrosis as defined in the 10th Edition (International Classification of Diseases, 10th Revision). Myelofibrosis exhibits an annual incidence of roughly 1 case per 100,000 individuals, typically diagnosed around the age of 60. The database included 1171 patients with essential hemorrhagic thrombocythemia and 344,335 controls (Table 1).

### 2.2. Instrumental variables (IVs) selection

Valid IVs are characterized by 3 fundamental assumptions.^[10]^ We screened variants associated with LDL at genome-wide significance (*P* < 5 × 10^−8^), defined by a linkage disequilibrium threshold of *r*^2^ < 0.1, using PLINK and phase 3 version 5 of the 1000 Genomes Project as a reference panel. These variants were located within ±100 kb of the 3-hydroxy-3-methylglutaryl-CoA reductase (*HMGCR*) gene (GRCh37/hg19: chromosome 5:74,632,154–74,657,929) for the purpose of instrumenting statins, the *NPC1L1* gene (chromosome 7: 44,552,134–44,580,914) for instrumenting ezetimibe, and the *PCSK9* gene (chromosome 1: 55,505,221–55,530,525) to instrument PCSK9 inhibitors, such as alirocumab or evolocumab. Figure 1 shows the hypothesis and study design.

Based on the observation that lipids are associated with primary hemorrhagic thrombocythemia, we investigated whether LDL levels are associated with the risk of primary (hemorrhagic) thrombocythemia rather than being a target of lipid-lowering drugs. We assayed significant genome-wide variation in LDL throughout the linkage disequilibrium clumping (*r*^2^ < 0.001 and clump window within 10,000 kilobases), excluding variation within the target gene regions of the 3 drugs mentioned above. To further mitigate the potential impact of pleiotropy and weak tools on our results, IVs with *F* statistics >10 were screened (Table S1, Supplemental Digital Content).

### 2.3. Statistical analysis

In our study, a variety of methods were applied to estimate causality and effects between exposures and outcomes, including inverse variance weighted, MR-Egger, weighted median, and weighted mode. The Wald ratio method was used to derive MR to estimate each single nucleotide polymorphism (SNP). In the presence of multiple SNPS, the inverse variance weighted method is used to calculate the weighted average of the ratio estimates, the weight of which is determined by the reciprocal variance of these estimates.^[10]^ In addition to assessing horizontal pleiotropy by evaluating whether the MR-Egger intercept is <0.05, we applied MR pleiotropy residual sum and outlier methods to test for and treat pleiotropy. The Cochran *Q* test was utilized to assess heterogeneity. Furthermore, false discovery rate-corrected *P* values were computed, considering a false discovery rate of <0.05 as significant.^[11]^

To address potential genetic confounding, we conducted Bayesian colocalization analyses. Occasionally, an SNP may reside within 2 or more gene regions. To mitigate this ambiguity, we stipulated that if an SNP contains genetic information for multiple genes, its effect on essential hemorrhagic thrombocythemia could be confounded by these different genes, and we used colocalization analysis to confirm that genes for essential hemorrhagic thrombocythemia and lipid-lowering drugs may share causal genetic variation. In short, to obtain significant MR results, we performed colocalization analyses of essential thrombocytopenia risk in lipid-lowering drugs and SNPs of positive genes within ±100 kb, P_1_ = 1 × 10^−4^, P_2_ = 1 × 10^−4^, and P_12_ = 1 × 10^−5^.^[12]^ The probability that a given SNP is associated with essential hemorrhagic thrombocythemia is denoted as P_1_, the probability that an SNP is related to an important lipid-lowering drug gene is denoted as P_2_, and the probability that an SNP is associated with both essential hemorrhagic thrombocythemia and a lipid-lowering drug gene is denoted as P_12_. We utilize posterior probability to evaluate the support for various hypotheses, ranging from PPH0 to PPH4: PPH0 indicates independence from any trait; PPH1 is linked to gene expression without influencing the risk of essential hemorrhagic thrombocythemia; PPH2 is connected to the risk of essential hemorrhagic thrombocythemia but not to gene expression; PPH3 is associated with both essential hemorrhagic thrombocythemia and gene expression, suggesting a definitive causal variant; and PPH4, which is related to both the risk of essential hemorrhagic thrombocythemia and gene expression, implies the existence of shared causal variants. Considering the limitations of colocalization analysis, we restricted further analysis to genes where the ratio PPH4/(PPH3 + PPH4) is ≥0.7.^[13]^

### 2.4. Supplementary analysis

To validate the findings of this study, we performed a series of complementary analyses to evaluate the robustness of our primary results. Considering the established efficacy of currently approved lipid-lowering drugs, we employed positive controls for coronary artery disease (CAD), where this methodology has been shown to be effective. We obtained the GWAS dataset on genetic correlates of CAD from the GWAS Catalog (https://www.ebi.ac.uk/gwas/), which included 60,801 clinically confirmed cases (including myocardial infarction, acute coronary atherosclerotic syndrome, chronic stable angina, or coronary artery stenosis >50%) and 123,504 controls^[14]^ (Table 1).

## 3. Results

**Figure 2.**
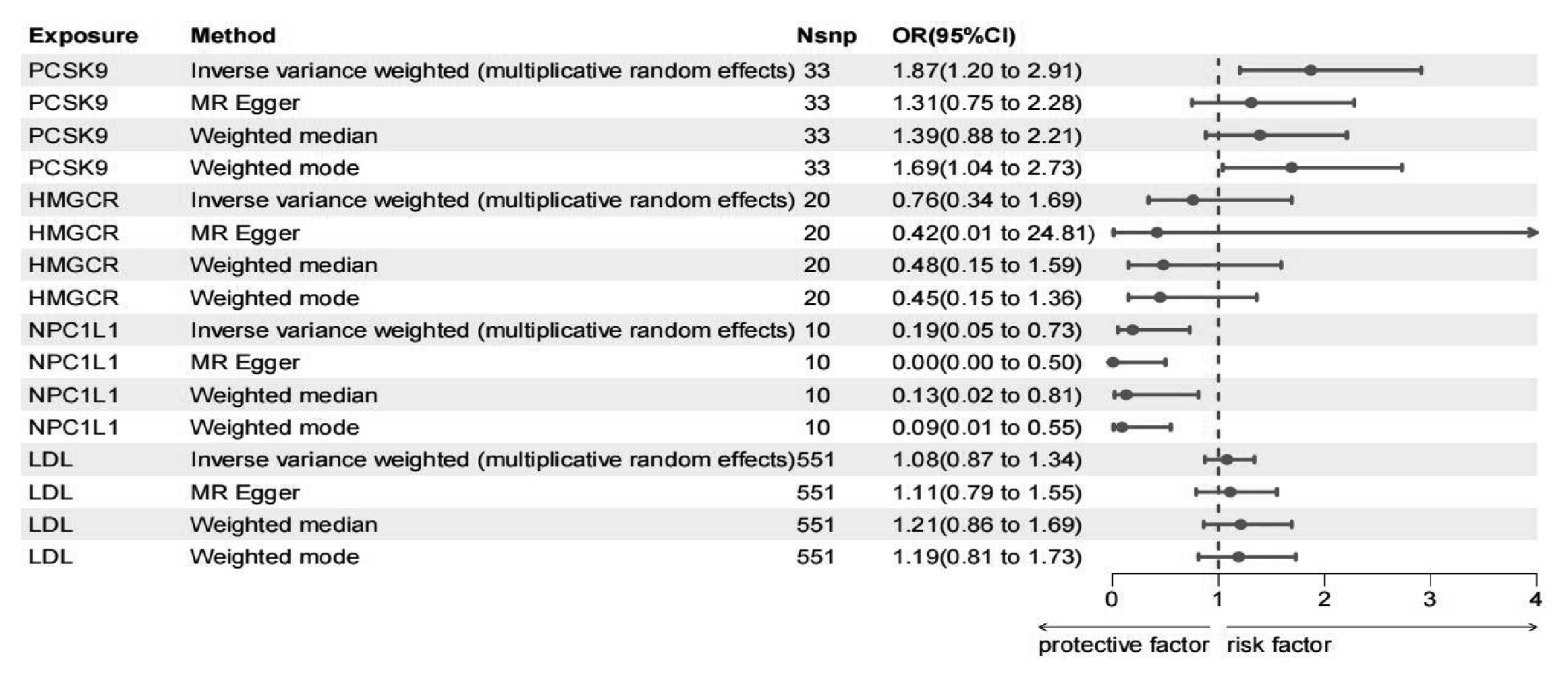
Summary of essential hemorrhagic thrombocythemia risk results from pleiotropy robust sensitivity analyses. CI = confidence interval, HMGCR = 3-hydroxy-3-methylglutaryl-CoA reductase, LDL = low-density lipoprotein, MR = Mendelian randomization, NPC1L1 = NPC1-like intracellular cholesterol transporter 1, OR = odds ratio, PCSK9 = proprotein convertase subtilisin/kexin type 9.

Analysis of FinnGen data identified 20 variants representing LDL reduction through HMGCR inhibition (mean *F*-statistic value of 175). Ten variants were identified for NPC1L1 inhibition, with an *F*-statistic value of 114, and 13 variants for PCSK9 inhibition, with an *F*-statistic value of 390. Genetically proxied PCSK9 inhibition was associated with an increased risk of essential hemorrhagic thrombocythemia (preponderance ratio of 1.87 per standard deviation LDL increased; 95% confidence interval [CI] = 1.2–2.91; *P* = .01), whereas genetically proxied NPC1L1 inhibition was linked to a reduced risk of essential hemorrhagic thrombocythemia (preponderance ratio of 0.19 per standard deviation LDL reduction; 95% CI = 0.05–0.73; *P* = .02). No risk association was found between HMGCR inhibition and essential hemorrhagic thrombocythemia (odds ratio [OR] = 0.76, 95% CI = 0.34–1.69, *P* = .51; Table S2, Supplemental Digital Content, and Fig. 2). Sensitivity analyses demonstrated consistent estimates and revealed no statistical evidence of bias due to horizontal pleiotropy (Fig. 2; Tables S2 and S3, Supplemental Digital Content). Within the FinnGen dataset, the PPH4/(PPH3 + PPH4) of colocalization between LDL and prothrombocytosis in the *PCSK9* gene region was 85%, and the *NPC1L1* gene region was 81% (Table S4, Supplemental Digital Content).

### 3.1. Supplementary analysis results

**Figure 3.**
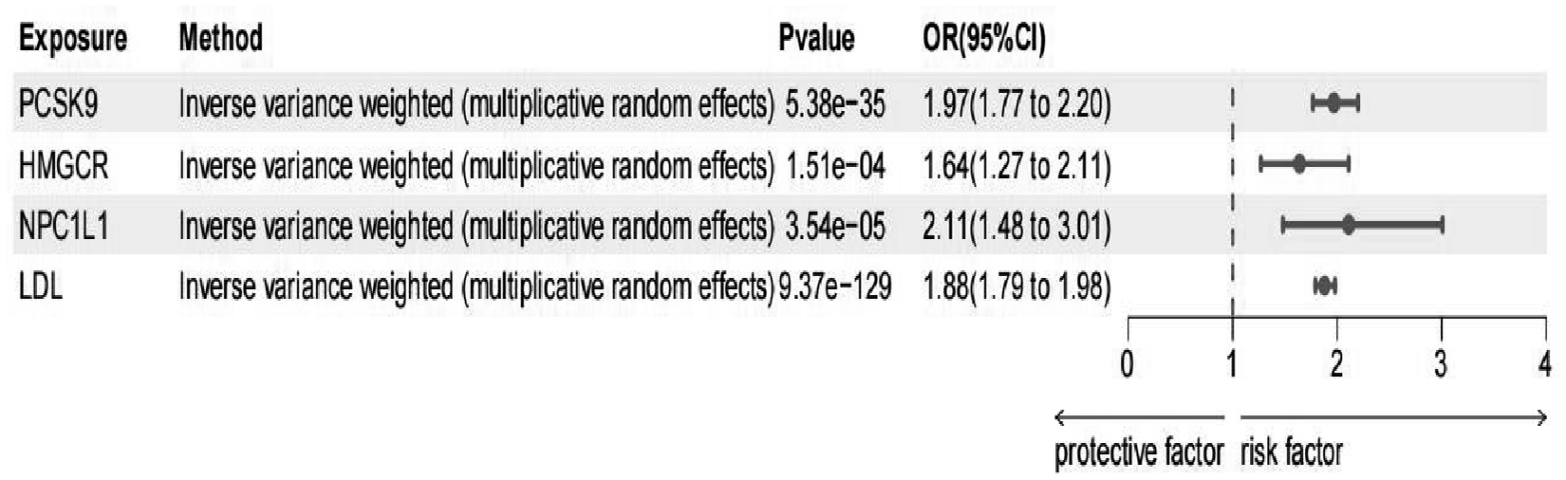
Associations between genetically proxied lipid-lowering drugs and risk of coronary artery disease as the positive control. CI = confidence interval, HMGCR = 3-hydroxy-3-methylglutaryl-CoA reductase, LDL = low-density lipoprotein, NPC1L1 = NPC1-like intracellular cholesterol transporter 1, OR = odds ratio, PCSK9 = proprotein convertase subtilisin/kexin type 9.

A reduction in CAD risk was associated with genetic inhibition of all 3 drug targets, including LDL: PCSK9 (OR = 1.97, 95% CI = 1.77–2.20, *P* = 1.08E-34), HMGCR (OR = 1.64, 95%CI = 1.27–2.21, *P* = 1.51E-4), and NPC1L1 (OR = 2.11, 95% CI = 1.48–3.01, *P* = 4.72E-5; Fig. 3 Tables S1 and S2, Supplemental Digital Content).

## 4. Discussion

Our MR study targeting drug effects demonstrated that, in a European population, inhibition of the *PCSK9* gene is causally linked to an increased risk of essential hemorrhagic thrombocythemia; however, *NPC1L1* gene inhibitors are associated with a reduced risk. This association does not appear to be related to circulating LDL levels, as we did not observe an overall association of LDL with essential hemorrhagic) thrombocythemia. The gene agent HMGCR (statin targeted) was not associated with essential hemorrhagic thrombocythemia risk.

There are several reasons for the possibility of studying disease modification through manipulation of the lipid pathway in essential hemorrhagic thrombocythemia. First, the pathogenesis of essential hemorrhagic thrombocythemia is not fully understood. Thus, uncovering the causal pathways involved in lipids may enhance our understanding of the mechanisms of progression in essential hemorrhagic thrombocythemia. Second, the selection of lipid-lowering agents with dual effects on lipids and essential hemorrhagic thrombocythemia can facilitate individualized treatment (for example, for patients with dyslipidemia and essential hemorrhagic thrombocythemia who require adjuvant therapy). Third, evidence that lipid-lowering drugs improve disease properties may also encourage their reintroduction into the treatment of essential hemorrhagic thrombocythemia.

Elevated concentrations of proprotein convertase PCSK9, a member of the serine protease enzyme family, have been linked to an elevated prevalence of cardiovascular disorders. Inhibitors targeting PCSK9 could potentially mitigate cardiovascular risks through the regulation of LDL receptors. These findings suggest that PCSK9 inhibitors may have a therapeutic role in managing cardiovascular disease by influencing LDL receptor function. This approach could offer a promising strategy for reducing the incidence of cardiovascular events.^[15]^ Recent studies have found that PCSK9 is expressed in the cardiovascular system and accelerates atherosclerosis. This effect is independent of the lipid regulation function and is regulated by multiple atherosclerosis-promoting mediators. A prospective study investigated the level of PCSK9 in patients with atrial fibrillation, indicating an interaction between PCSK9 and platelet activation.^[16]^ There is evidence that PCSK9 promotes platelet aggregation, activation, and proliferation through interaction with CD36 on the platelet surface.^[17,18]^ PCSK9 regulates LDL receptor recycling and promotes platelet activation via CD36 signaling. Inhibitors (e.g., alirocumab) reduce LDL and may attenuate platelet hyperreactivity by decreasing membrane cholesterol density.^[19]^ This dual cardioprotective and antiplatelet action suggests translational potential for bleeding-prone ET patients, particularly those with cardiovascular comorbidities. Collectively, these findings offer compelling evidence linking PCSK9 to the pathophysiology of essential hemorrhagic thrombocythemia. PCSK9 could be a target for the treatment of the disease.

NPC1L1 is a transmembrane protein, a molecular target of ezetimibe.^[20]^ It is a target of commonly used clinical lipid-lowering drugs. NPC1L1 inhibition (e.g., ezetimibe) reduces intestinal cholesterol absorption and may modulate platelet function by altering lipid raft composition. Pharmacologically, ezetimibe’s minimal systemic absorption favors localized effects, potentially mitigating hemorrhagic risk without exacerbating thrombocytopenia – a critical consideration in ET management. A study on the effect of NPC1L1 on early-onset 3-vessel CAD showed that certain alleles of NPC1L1 increase coronary artery stenosis. It provides a rationale for drug development.^[21]^ Essential hemorrhagic thrombocythemia is primarily associated with coagulation mechanisms. A clinical study of *NPC1L1* gene polymorphisms affecting warfarin dosing suggests that mutations at certain loci of the *NPC1L1* gene affect warfarin metabolism. This in turn affects patient coagulation.^[22]^ To our knowledge, research on NPC1L1 and essential hemorrhagic thrombocythemia has been limited. However, due to the lack of relevant animal and clinical observational studies, this MR study may offer guidance for future investigations into NPC1L1-related pathways and the prevention and treatment of essential hemorrhagic thrombocythemia.

Statins are usually used as lipid-lowering agents to lower cholesterol levels by inhibiting HMGCR.^[23]^ In addition to lipid-lowering, statins have been shown to modulate inflammatory cytokines and endothelial function.^[24]^ Statins’ antiplatelet effects are largely attributed to pleiotropic Rho/Rho-kinase inhibition rather than HMGCR blockade per se. Our MR estimates reflect genetic inhibition of HMGCR, which may not capture statins’ pleiotropic signaling effects. Statins reduce circulating levels of soluble CD40, which in turn reduces platelet action in patients with chronic obstructive pulmonary disease.^[25]^ Consequently, a CD40-mediated pro-thrombotic state is also present.^[26–30]^ Recently, atorvastatin has been shown to reduce platelet activity and LDL receptor changes in patients with hypercholesterolemia by reducing the level of oxidized protein. Platelet inactivation occurs prior to significant changes in LDL levels. Consequently, the antiatherosclerotic effect of atorvastatin is thought to reduce platelet activation and platelet lesion by regulating the expression of oxidized LDL, which has been shown to enhance the efficacy of statins in essential hemorrhagic thrombocythemia. However, current MR analyses have found no evidence to support a causal relationship between HMGCR suppression and essential platelet risk. It may be that ET patients exhibit abnormal JAK2-mediated megakaryopoiesis that may override HMGCR-related platelet regulation. In contrast, the efficacy of statins in cardiovascular cohorts may reflect synergy with endothelial dysfunction – a less prominent feature in ET. Second, clinical studies reporting platelet inhibition by statins typically involve high doses/long-term exposures. MR represents lifelong HMGCR inhibition at the physiologic level, which may not be sufficient to trigger platelet regulation.

The main strength of this study is the stability of the results. There is more favorable evidence than traditional observational studies. We use a variety of methods to test the robustness of the results and strictly adhere to the 3 main assumptions of MR when screening variables. In addition, our study used MR analysis, drawing on data from recent Finnish and global genomic studies, with a larger sample size and better control for confounders, improving the reliability of causal reasoning. This MR study has several limitations. First, risk factors for disease onset may differ from those affecting disease severity or prognosis, meaning our findings primarily address the treatment of essential hemorrhagic thrombocythemia. However, related studies indicate potential therapeutic benefits in treating essential hemorrhagic thrombocythemia. Second, MR estimates should not be directly compared with pharmacological inhibition, as subtle variations in PCSK9 levels due to genetic differences do not directly reflect the effects of drug-based inhibition. Moreover, lifelong exposure through MR is distinct from the short-term effects of pharmacological interventions. In addition, genetic variations in PCSK9 across the body may not align with the specific tissue targets of pharmacological treatments.^[31]^ MR analysis indicates a causal relationship between PCSK9 inhibition and a decreased risk of essential hemorrhagic thrombocythemia, although relevant animal experiments and clinical observational studies are currently lacking. These studies may, in the future, provide evidence for the association between PCSK9 inhibition and essential hemorrhagic thrombocythemia pathways, as well as inform drug prevention and treatment strategies. Third, the group of people we studied only included those who are from Europe. We need to do more research with people from different backgrounds to see if our findings apply to everyone. We also need to look at data from a bigger study to make sure our results are correct.

## 5. Conclusions

Our study suggests that NPC1L1 inhibition is causally associated with a decrease in primary hemorrhagic thrombocytopenia, whereas PSCK9 inhibition increases the risk of this disease. Nonetheless, additional studies are required to evaluate the feasibility of these targets as therapeutic agents for essential hemorrhagic thrombocythemia. Simultaneously, caution is warranted when formulating therapeutic recommendations based on MR analysis results, as these findings require validation through clinical trials.

## Acknowledgments

The authors sincerely thank related investigators for sharing the statistics included in this study.

## Author contributions

**Conceptualization:** Peng Zhang, Zheng Nan.

**Data curation:** Peng Zhang, HaiJiao Wang, MingHao Lin, Peng Dai, Qi Zhong, RuoLin Wang, Lei Sheng.

**Funding acquisition:** Peng Zhang.

**Investigation:** Peng Zhang, Lei Sheng.

**Methodology:** Peng Zhang, Peng Dai.

**Project administration:** Peng Zhang, HaiJiao Wang, Ze He.

**Software:** Peng Zhang.

**Supervision:** HaiJiao Wang.

**Resources:** RuoLin Wang.

**Writing – original draft:** Ze He.

**Writing – review & editing:** Ze He.










