# Supplementary material for: Lipid-lowering drugs and essential hemorrhagic thrombocythemia’s risk: A drug-target Mendelian randomization study
Source: Medicine (Baltimore). 2026 May 29;105(22):e49077. doi: 10.1097/MD.0000000000049077 (PMC13225517; doi:10.1097/MD.0000000000049077)
Supplement: Supplementary file 2 [file medi-105-e49077-s002.docx]

| **Table S1. Genetic variants used to instrument LDL and each lipid lowering drug target for the primary analysis.** | | | | | | | |
| --- | --- | --- | --- | --- | --- | --- | --- |
|  |  |  |  |  |  |  |  |
|  |  |  |  |  |  |  |  |
| **Exposure** | **SNP** | **EA/OA** | **EAF** | **beta** | **se** | **p--al** | **F** |
| HMGCR | rs375392181 | T/G | 0.01 | 0.046 | 0.007 | 1.53E-10 | 41 |
| HMGCR | rs17738989 | T/C | 0.365 | 0.047 | 0.002 | 1.00E-200 | 976 |
| HMGCR | rs1308220 | G/A | 0.033 | 0.049 | 0.005 | 1.48E-23 | 100 |
| HMGCR | rs146795953 | A/G | 0.009 | -0.057 | 0.01 | 2.81E-08 | 31 |
| HMGCR | rs115845757 | A/G | 0.019 | 0.06 | 0.005 | 5.14E-28 | 120 |
| HMGCR | rs4703665 | T/C | 0.138 | -0.03 | 0.002 | 6.57E-38 | 166 |
| HMGCR | rs35122945 | C/A | 0.066 | -0.036 | 0.003 | 6.73E-32 | 138 |
| HMGCR | rs151214061 | C/T | 0.022 | 0.043 | 0.005 | 3.21E-18 | 76 |
| HMGCR | rs6895057 | A/G | 0.243 | 0.046 | 0.002 | 2.52E-173 | 788 |
| HMGCR | rs75240579 | T/C | 0.041 | -0.041 | 0.004 | 2.27E-27 | 117 |
| HMGCR | rs112672253 | T/A | 0.008 | 0.061 | 0.008 | 4.73E-13 | 52 |
| HMGCR | rs17244939 | C/A | 0.016 | -0.043 | 0.006 | 1.28E-11 | 46 |
| HMGCR | rs115169875 | A/G | 0.026 | -0.033 | 0.005 | 1.22E-12 | 50 |
| HMGCR | rs62366588 | A/C | 0.058 | -0.035 | 0.003 | 9.30E-26 | 110 |
| HMGCR | rs74695562 | G/T | 0.042 | -0.036 | 0.004 | 8.63E-22 | 92 |
| HMGCR | rs17562727 | C/T | 0.027 | 0.048 | 0.005 | 4.04E-25 | 107 |
| HMGCR | rs144083983 | T/C | 0.07 | -0.042 | 0.003 | 3.23E-41 | 181 |
| HMGCR | rs181668591 | T/C | 0.01 | 0.06 | 0.007 | 1.75E-15 | 63 |
| HMGCR | rs114253542 | C/T | 0.008 | 0.076 | 0.009 | 4.06E-18 | 75 |
| HMGCR | rs180755046 | A/C | 0.016 | -0.041 | 0.006 | 1.11E-10 | 42 |
| HMGCR | rs182826525 | G/A | 0.014 | 0.101 | 0.009 | 2.67E-30 | 131 |
| HMGCR | rs151000110 | A/G | 0.056 | 0.068 | 0.003 | 6.18E-99 | 446 |
| HMGCR | rs200823803 | C/T | 0.018 | 0.055 | 0.006 | 4.18E-19 | 80 |
| NPC1L1 | rs141530948 | A/G | 0.006 | -0.057 | 0.009 | 2.19E-09 | 36 |
| NPC1L1 | rs77826622 | C/T | 0.023 | 0.032 | 0.005 | 1.32E-10 | 41 |
| NPC1L1 | rs7808295 | T/C | 0.041 | -0.022 | 0.004 | 5.93E-09 | 34 |
| NPC1L1 | rs17725246 | C/T | 0.189 | 0.043 | 0.002 | 2.29E-116 | 526 |
| NPC1L1 | rs77517259 | T/C | 0.008 | 0.053 | 0.008 | 2.83E-10 | 40 |
| NPC1L1 | rs217370 | G/A | 0.468 | -0.025 | 0.001 | 2.35E-69 | 310 |
| NPC1L1 | rs79836087 | A/G | 0.046 | -0.021 | 0.003 | 1.73E-09 | 36 |
| NPC1L1 | rs79854399 | T/C | 0.018 | -0.037 | 0.006 | 4.53E-11 | 43 |
| NPC1L1 | rs143116067 | A/G | 0.068 | -0.02 | 0.003 | 1.49E-11 | 46 |
| NPC1L1 | rs17133653 | C/G | 0.023 | 0.027 | 0.005 | 8.41E-09 | 33 |
| PCSK9 | rs12043403 | C/T | 0.096 | -0.016 | 0.003 | 3.43E-10 | 39 |
| PCSK9 | rs11206498 | G/A | 0.342 | 0.009 | 0.002 | 8.01E-10 | 38 |
| PCSK9 | rs890576 | G/C | 0.201 | -0.017 | 0.002 | 1.15E-20 | 87 |
| PCSK9 | rs146273942 | A/G | 0.021 | -0.049 | 0.005 | 2.00E-21 | 90 |
| PCSK9 | rs17111474 | T/C | 0.298 | 0.011 | 0.002 | 1.90E-10 | 41 |
| PCSK9 | rs77875082 | A/G | 0.032 | 0.054 | 0.004 | 4.99E-37 | 162 |
| PCSK9 | rs12117661 | G/C | 0.243 | -0.064 | 0.002 | 1.00E-200 | 1435 |
| PCSK9 | rs374459115 | A/G | 0.018 | 0.069 | 0.007 | 8.80E-24 | 101 |
| PCSK9 | rs28775984 | C/T | 0.433 | -0.02 | 0.003 | 6.25E-11 | 43 |
| PCSK9 | rs2479420 | C/T | 0.262 | 0.036 | 0.002 | 7.46E-92 | 413 |
| PCSK9 | rs182491400 | T/C | 0.022 | 0.043 | 0.006 | 2.02E-14 | 59 |
| PCSK9 | rs181331606 | G/C | 0.09 | 0.028 | 0.003 | 1.94E-20 | 86 |
| PCSK9 | rs142791622 | A/G | 0.023 | -0.062 | 0.005 | 3.35E-37 | 162 |
| PCSK9 | rs80085738 | A/G | 0.011 | -0.041 | 0.007 | 2.87E-09 | 35 |
| PCSK9 | rs12739979 | T/C | 0.24 | -0.024 | 0.002 | 1.24E-37 | 164 |
| PCSK9 | rs72660548 | G/C | 0.019 | 0.073 | 0.005 | 6.09E-44 | 193 |
| PCSK9 | rs17111503 | G/A | 0.259 | 0.048 | 0.002 | 1.44E-188 | 858 |
| PCSK9 | rs2479408 | G/C | 0.2 | -0.04 | 0.002 | 4.51E-106 | 479 |
| PCSK9 | rs11591147 | T/G | 0.015 | -0.427 | 0.006 | 1.00E-200 | 5475 |
| PCSK9 | rs28385706 | C/A | 0.016 | -0.04 | 0.006 | 2.83E-12 | 49 |
| PCSK9 | rs479832 | C/T | 0.18 | -0.015 | 0.002 | 1.44E-14 | 59 |
| PCSK9 | rs2495477 | G/A | 0.395 | -0.05 | 0.002 | 1.00E-200 | 1064 |
| PCSK9 | rs41294825 | T/A | 0.044 | -0.041 | 0.003 | 6.08E-33 | 143 |
| PCSK9 | rs7525503 | T/G | 0.023 | 0.072 | 0.005 | 2.68E-44 | 195 |
| PCSK9 | rs11206517 | G/T | 0.037 | 0.088 | 0.004 | 5.47E-117 | 529 |
| PCSK9 | rs41297885 | G/C | 0.036 | -0.041 | 0.004 | 3.95E-26 | 112 |
| PCSK9 | rs28385715 | G/T | 0.023 | 0.046 | 0.005 | 3.75E-20 | 85 |
| PCSK9 | rs77011887 | T/C | 0.017 | 0.046 | 0.006 | 2.21E-15 | 63 |
| PCSK9 | rs142116310 | A/G | 0.011 | 0.055 | 0.008 | 2.04E-13 | 54 |
| PCSK9 | rs10493176 | G/T | 0.08 | -0.066 | 0.003 | 7.55E-135 | 611 |
| PCSK9 | rs115465289 | A/G | 0.032 | -0.041 | 0.004 | 3.96E-22 | 94 |
| PCSK9 | rs12031153 | A/G | 0.052 | 0.034 | 0.004 | 1.67E-20 | 86 |
| PCSK9 | rs79494709 | C/G | 0.05 | -0.027 | 0.003 | 1.11E-15 | 64 |
| PCSK9 | rs55817205 | A/G | 0.012 | 0.069 | 0.007 | 3.91E-21 | 89 |
| LDL | rs1123571 | A/G | 0.466 | -0.011 | 0.001 | 1.26E-13 | 55 |
| LDL | rs1497406 | A/G | 0.42 | -0.013 | 0.001 | 3.36E-20 | 85 |
| LDL | rs2992752 | A/C | 0.371 | 0.011 | 0.001 | 5.52E-13 | 52 |
| LDL | rs11578642 | A/G | 0.167 | 0.015 | 0.002 | 1.23E-13 | 55 |
| LDL | rs7551124 | C/T | 0.126 | -0.017 | 0.002 | 4.63E-15 | 61 |
| LDL | rs10903129 | A/G | 0.452 | -0.025 | 0.001 | 1.25E-69 | 311 |
| LDL | rs12094989 | T/C | 0.214 | -0.012 | 0.002 | 5.89E-12 | 47 |
| LDL | rs114165349 | C/G | 0.023 | 0.082 | 0.005 | 1.30E-64 | 288 |
| LDL | rs59669045 | A/G | 0.25 | -0.01 | 0.002 | 3.83E-09 | 35 |
| LDL | rs4329504 | G/A | 0.419 | -0.008 | 0.001 | 3.20E-08 | 31 |
| LDL | rs638769 | G/C | 0.295 | -0.009 | 0.002 | 5.86E-09 | 34 |
| LDL | rs17853159 | A/G | 0.078 | -0.022 | 0.003 | 1.06E-16 | 69 |
| LDL | rs72677559 | G/C | 0.2 | 0.01 | 0.002 | 8.34E-09 | 33 |
| LDL | rs72660594 | C/T | 0.013 | -0.198 | 0.006 | 1.00E-200 | 933 |
| LDL | rs1475701 | C/T | 0.037 | 0.087 | 0.004 | 5.81E-113 | 510 |
| LDL | rs146346813 | C/A | 0.027 | -0.059 | 0.005 | 4.51E-39 | 171 |
| LDL | rs148742777 | C/T | 0.011 | 0.063 | 0.008 | 3.25E-16 | 67 |
| LDL | rs12750160 | T/C | 0.009 | -0.303 | 0.008 | 1.00E-200 | 1455 |
| LDL | rs11803234 | T/C | 0.044 | -0.036 | 0.003 | 4.32E-25 | 107 |
| LDL | rs79030606 | A/G | 0.032 | -0.035 | 0.004 | 2.80E-17 | 71 |
| LDL | rs150088310 | A/G | 0.015 | 0.043 | 0.006 | 6.28E-13 | 52 |
| LDL | rs17457613 | C/G | 0.021 | -0.049 | 0.005 | 5.21E-21 | 88 |
| LDL | rs113654127 | A/G | 0.013 | -0.041 | 0.007 | 2.79E-10 | 40 |
| LDL | rs11806791 | C/A | 0.077 | -0.018 | 0.003 | 3.22E-11 | 44 |
| LDL | rs7534572 | C/G | 0.33 | -0.042 | 0.002 | 3.19E-163 | 741 |
| LDL | rs6684364 | A/G | 0.249 | -0.009 | 0.002 | 1.17E-08 | 33 |
| LDL | rs2391159 | T/C | 0.206 | -0.023 | 0.002 | 3.14E-38 | 167 |
| LDL | rs192937664 | T/C | 0.018 | -0.035 | 0.006 | 2.97E-09 | 35 |
| LDL | rs111232683 | C/G | 0.344 | 0.014 | 0.002 | 1.36E-16 | 68 |
| LDL | rs4970824 | A/G | 0.068 | -0.088 | 0.003 | 1.00E-200 | 943 |
| LDL | rs114713488 | C/A | 0.028 | 0.038 | 0.004 | 6.28E-19 | 79 |
| LDL | rs149841512 | A/G | 0.023 | 0.042 | 0.005 | 1.99E-16 | 68 |
| LDL | rs6670347 | C/T | 0.04 | -0.109 | 0.004 | 3.06E-194 | 884 |
| LDL | rs17036094 | C/A | 0.012 | -0.115 | 0.006 | 1.28E-70 | 316 |
| LDL | rs148631275 | C/T | 0.018 | 0.035 | 0.006 | 9.76E-10 | 37 |
| LDL | rs10858093 | T/C | 0.026 | 0.046 | 0.005 | 2.05E-20 | 86 |
| LDL | rs149516707 | G/C | 0.021 | -0.045 | 0.005 | 1.52E-17 | 73 |
| LDL | rs115458560 | C/T | 0.016 | -0.049 | 0.006 | 1.23E-16 | 69 |
| LDL | rs657801 | C/T | 0.315 | 0.01 | 0.002 | 3.20E-10 | 40 |
| LDL | rs267733 | G/A | 0.156 | -0.018 | 0.002 | 1.49E-20 | 86 |
| LDL | rs113462784 | C/T | 0.121 | 0.015 | 0.003 | 3.34E-08 | 30 |
| LDL | rs4390169 | A/G | 0.485 | 0.012 | 0.001 | 5.40E-16 | 66 |
| LDL | rs12045893 | T/C | 0.246 | 0.011 | 0.002 | 1.27E-10 | 41 |
| LDL | rs115383270 | A/G | 0.074 | 0.02 | 0.003 | 1.19E-12 | 51 |
| LDL | rs2933547 | G/A | 0.414 | 0.008 | 0.001 | 3.02E-08 | 31 |
| LDL | rs76900682 | A/G | 0.153 | 0.013 | 0.002 | 5.17E-10 | 39 |
| LDL | rs6682862 | A/G | 0.164 | -0.014 | 0.002 | 6.82E-13 | 52 |
| LDL | rs1779807 | T/C | 0.328 | 0.014 | 0.002 | 1.01E-21 | 92 |
| LDL | rs2296288 | T/C | 0.438 | -0.011 | 0.001 | 2.57E-14 | 58 |
| LDL | rs1434282 | C/T | 0.276 | -0.011 | 0.002 | 3.37E-11 | 44 |
| LDL | rs2642438 | A/G | 0.295 | -0.027 | 0.002 | 2.46E-64 | 287 |
| LDL | rs12117480 | A/C | 0.038 | -0.025 | 0.004 | 2.74E-11 | 44 |
| LDL | rs7544869 | A/T | 0.451 | -0.012 | 0.001 | 1.26E-17 | 73 |
| LDL | rs553427 | C/T | 0.474 | -0.04 | 0.001 | 3.42E-169 | 769 |
| LDL | rs10910522 | A/G | 0.43 | -0.012 | 0.001 | 8.42E-16 | 65 |
| LDL | rs7512010 | A/T | 0.195 | -0.019 | 0.002 | 1.58E-24 | 104 |
| LDL | rs3935011 | C/T | 0.485 | 0.009 | 0.001 | 9.55E-10 | 37 |
| LDL | rs3820897 | T/C | 0.185 | 0.016 | 0.002 | 6.91E-19 | 79 |
| LDL | rs67269656 | T/C | 0.262 | -0.01 | 0.002 | 7.08E-10 | 38 |
| LDL | rs7556983 | A/G | 0.105 | -0.015 | 0.002 | 8.50E-11 | 42 |
| LDL | rs6734686 | C/T | 0.209 | 0.015 | 0.002 | 3.05E-16 | 67 |
| LDL | rs141481562 | G/A | 0.007 | 0.074 | 0.01 | 7.70E-14 | 56 |
| LDL | rs75980706 | T/G | 0.019 | -0.034 | 0.005 | 2.05E-10 | 40 |
| LDL | rs11096689 | C/T | 0.293 | 0.047 | 0.002 | 1.00E-200 | 914 |
| LDL | rs35239705 | A/G | 0.191 | -0.064 | 0.002 | 1.00E-200 | 1187 |
| LDL | rs113311561 | A/G | 0.009 | -0.065 | 0.01 | 1.35E-11 | 46 |
| LDL | rs994286 | A/G | 0.02 | -0.034 | 0.005 | 4.89E-10 | 39 |
| LDL | rs112128540 | C/G | 0.034 | 0.033 | 0.004 | 1.24E-14 | 59 |
| LDL | rs11125502 | C/T | 0.286 | 0.009 | 0.002 | 8.26E-09 | 33 |
| LDL | rs142787485 | G/A | 0.037 | -0.029 | 0.004 | 1.39E-12 | 50 |
| LDL | rs1731243 | C/T | 0.399 | -0.009 | 0.001 | 3.03E-10 | 40 |
| LDL | rs1260326 | T/C | 0.392 | 0.03 | 0.001 | 1.55E-97 | 439 |
| LDL | rs71441185 | G/A | 0.03 | 0.024 | 0.004 | 1.38E-08 | 32 |
| LDL | rs150893163 | A/T | 0.02 | 0.03 | 0.005 | 1.12E-08 | 33 |
| LDL | rs10495907 | A/G | 0.137 | 0.03 | 0.002 | 2.91E-46 | 204 |
| LDL | rs56266464 | A/G | 0.06 | -0.112 | 0.003 | 1.00E-200 | 1374 |
| LDL | rs191243200 | A/G | 0.013 | 0.066 | 0.006 | 8.05E-25 | 106 |
| LDL | rs35206901 | G/A | 0.184 | -0.041 | 0.002 | 2.52E-108 | 489 |
| LDL | rs1811515 | C/G | 0.394 | -0.009 | 0.001 | 9.15E-10 | 37 |
| LDL | rs12712955 | A/G | 0.496 | 0.011 | 0.001 | 4.88E-15 | 61 |
| LDL | rs77871695 | A/C | 0.141 | -0.012 | 0.002 | 4.61E-09 | 34 |
| LDL | rs4671050 | T/G | 0.318 | -0.022 | 0.002 | 1.57E-45 | 201 |
| LDL | rs13023873 | T/C | 0.15 | -0.015 | 0.002 | 1.69E-13 | 54 |
| LDL | rs10193634 | C/A | 0.392 | -0.012 | 0.001 | 3.19E-15 | 62 |
| LDL | rs964392 | G/A | 0.398 | 0.011 | 0.001 | 1.26E-13 | 55 |
| LDL | rs11887443 | T/G | 0.265 | -0.012 | 0.002 | 5.06E-12 | 48 |
| LDL | rs2970902 | C/G | 0.338 | 0.01 | 0.002 | 9.27E-12 | 46 |
| LDL | rs10185855 | G/A | 0.362 | -0.013 | 0.001 | 7.28E-18 | 74 |
| LDL | rs2718702 | T/C | 0.192 | 0.019 | 0.002 | 1.65E-25 | 109 |
| LDL | rs55709272 | C/T | 0.444 | -0.009 | 0.001 | 1.74E-10 | 41 |
| LDL | rs116704418 | A/G | 0.056 | -0.019 | 0.003 | 9.61E-10 | 37 |
| LDL | rs141213470 | T/A | 0.015 | 0.045 | 0.006 | 2.81E-13 | 53 |
| LDL | rs150474434 | A/G | 0.096 | -0.042 | 0.002 | 3.78E-67 | 300 |
| LDL | rs552636263 | T/C | 0.018 | 0.035 | 0.006 | 2.58E-08 | 31 |
| LDL | rs17050272 | A/G | 0.416 | -0.024 | 0.001 | 1.00E-56 | 252 |
| LDL | rs1375131 | C/T | 0.308 | 0.02 | 0.002 | 9.61E-28 | 119 |
| LDL | rs13014455 | C/T | 0.237 | 0.01 | 0.002 | 2.25E-08 | 31 |
| LDL | rs12614487 | T/C | 0.075 | -0.027 | 0.003 | 6.57E-23 | 97 |
| LDL | rs10184004 | T/C | 0.416 | -0.01 | 0.001 | 1.36E-12 | 50 |
| LDL | rs2389606 | C/T | 0.397 | 0.022 | 0.001 | 2.89E-51 | 227 |
| LDL | rs4129011 | C/T | 0.344 | -0.009 | 0.002 | 6.40E-09 | 34 |
| LDL | rs12693968 | A/G | 0.256 | 0.022 | 0.002 | 2.05E-39 | 173 |
| LDL | rs62182788 | T/C | 0.411 | -0.014 | 0.001 | 2.38E-21 | 90 |
| LDL | rs1250259 | T/A | 0.267 | -0.017 | 0.002 | 1.71E-26 | 113 |
| LDL | rs34353396 | C/T | 0.408 | 0.01 | 0.002 | 9.86E-10 | 37 |
| LDL | rs78058190 | A/G | 0.052 | 0.022 | 0.004 | 8.23E-09 | 33 |
| LDL | rs116001967 | A/G | 0.013 | -0.036 | 0.007 | 3.95E-08 | 30 |
| LDL | rs6431630 | A/G | 0.105 | 0.024 | 0.002 | 4.34E-26 | 112 |
| LDL | rs146194062 | T/C | 0.012 | -0.071 | 0.007 | 1.11E-22 | 96 |
| LDL | rs13076933 | G/T | 0.257 | -0.025 | 0.002 | 8.42E-51 | 225 |
| LDL | rs6792725 | A/G | 0.312 | 0.015 | 0.002 | 5.86E-21 | 88 |
| LDL | rs9837622 | A/T | 0.073 | -0.038 | 0.003 | 9.85E-43 | 188 |
| LDL | rs3755799 | A/G | 0.339 | 0.011 | 0.002 | 1.09E-12 | 51 |
| LDL | rs13066351 | T/C | 0.081 | -0.037 | 0.003 | 4.33E-47 | 208 |
| LDL | rs55921103 | G/T | 0.357 | -0.013 | 0.002 | 1.22E-16 | 69 |
| LDL | rs7637250 | G/C | 0.343 | 0.009 | 0.002 | 1.67E-08 | 32 |
| LDL | rs3732359 | G/A | 0.226 | 0.015 | 0.002 | 7.16E-19 | 79 |
| LDL | rs12636106 | T/C | 0.156 | 0.019 | 0.002 | 5.98E-23 | 97 |
| LDL | rs2011442 | C/T | 0.336 | -0.012 | 0.002 | 3.16E-15 | 62 |
| LDL | rs9862203 | A/G | 0.239 | -0.014 | 0.002 | 6.70E-16 | 65 |
| LDL | rs62264113 | A/G | 0.109 | 0.015 | 0.002 | 1.18E-10 | 41 |
| LDL | rs56299595 | G/A | 0.127 | 0.018 | 0.002 | 2.14E-17 | 72 |
| LDL | rs77436593 | A/G | 0.086 | -0.033 | 0.003 | 1.38E-37 | 164 |
| LDL | rs147810458 | A/G | 0.007 | -0.06 | 0.009 | 5.04E-11 | 43 |
| LDL | rs113322334 | C/T | 0.013 | -0.048 | 0.007 | 7.37E-13 | 51 |
| LDL | rs537321744 | T/C | 0.026 | -0.028 | 0.005 | 2.08E-08 | 31 |
| LDL | rs6794370 | A/C | 0.203 | 0.011 | 0.002 | 1.43E-08 | 32 |
| LDL | rs28478252 | A/C | 0.242 | -0.013 | 0.002 | 3.15E-15 | 62 |
| LDL | rs9653945 | A/G | 0.346 | -0.013 | 0.001 | 2.53E-17 | 72 |
| LDL | rs407258 | C/T | 0.251 | 0.01 | 0.002 | 6.00E-09 | 34 |
| LDL | rs4679881 | T/C | 0.466 | 0.013 | 0.001 | 8.34E-19 | 78 |
| LDL | rs56118251 | G/A | 0.159 | 0.013 | 0.002 | 3.99E-12 | 48 |
| LDL | rs16861497 | T/G | 0.391 | -0.009 | 0.001 | 4.60E-09 | 34 |
| LDL | rs2342307 | A/G | 0.377 | 0.009 | 0.002 | 8.00E-10 | 38 |
| LDL | rs13108218 | A/G | 0.388 | 0.019 | 0.002 | 2.49E-36 | 158 |
| LDL | rs4689653 | T/G | 0.383 | -0.012 | 0.001 | 5.54E-15 | 61 |
| LDL | rs2702571 | T/A | 0.367 | 0.009 | 0.001 | 7.93E-09 | 33 |
| LDL | rs6448432 | A/G | 0.297 | 0.009 | 0.002 | 9.16E-09 | 33 |
| LDL | rs12504134 | T/C | 0.34 | -0.009 | 0.002 | 6.43E-09 | 34 |
| LDL | rs76785150 | T/C | 0.067 | -0.018 | 0.003 | 2.36E-10 | 40 |
| LDL | rs11736427 | T/A | 0.364 | -0.008 | 0.001 | 3.92E-08 | 30 |
| LDL | rs112575086 | T/C | 0.121 | -0.013 | 0.002 | 1.07E-09 | 37 |
| LDL | rs11133477 | G/A | 0.212 | 0.011 | 0.002 | 5.03E-10 | 39 |
| LDL | rs34707604 | C/T | 0.248 | 0.031 | 0.002 | 2.02E-58 | 260 |
| LDL | rs13112099 | G/T | 0.469 | 0.011 | 0.002 | 2.42E-13 | 54 |
| LDL | rs28375964 | T/C | 0.485 | 0.009 | 0.001 | 4.96E-10 | 39 |
| LDL | rs72663045 | G/T | 0.02 | 0.041 | 0.005 | 2.15E-15 | 63 |
| LDL | rs71607360 | C/A | 0.087 | -0.015 | 0.003 | 4.70E-09 | 34 |
| LDL | rs3775228 | T/C | 0.395 | 0.01 | 0.001 | 1.21E-12 | 50 |
| LDL | rs2165670 | A/G | 0.11 | 0.016 | 0.002 | 2.23E-12 | 49 |
| LDL | rs28497720 | T/C | 0.253 | -0.017 | 0.002 | 3.69E-26 | 112 |
| LDL | rs13107325 | T/C | 0.067 | -0.025 | 0.003 | 2.66E-19 | 81 |
| LDL | rs17617028 | A/G | 0.215 | 0.013 | 0.002 | 1.44E-14 | 59 |
| LDL | rs10021804 | G/A | 0.374 | -0.009 | 0.001 | 3.08E-10 | 40 |
| LDL | rs138204164 | G/C | 0.13 | -0.014 | 0.002 | 3.16E-11 | 44 |
| LDL | rs35619904 | C/T | 0.372 | -0.008 | 0.001 | 1.44E-08 | 32 |
| LDL | rs2085723 | G/T | 0.355 | -0.01 | 0.001 | 3.30E-12 | 49 |
| LDL | rs41280463 | A/G | 0.166 | -0.015 | 0.002 | 2.12E-14 | 58 |
| LDL | rs72969958 | T/G | 0.009 | 0.063 | 0.008 | 1.17E-15 | 64 |
| LDL | rs72701754 | T/A | 0.404 | 0.009 | 0.001 | 1.54E-10 | 41 |
| LDL | rs116734477 | T/C | 0.038 | -0.052 | 0.004 | 3.51E-43 | 190 |
| LDL | rs112838464 | A/G | 0.109 | 0.013 | 0.002 | 3.22E-09 | 35 |
| LDL | rs13173241 | A/G | 0.197 | 0.015 | 0.002 | 4.08E-18 | 75 |
| LDL | rs2434583 | C/A | 0.467 | 0.008 | 0.001 | 4.04E-08 | 30 |
| LDL | rs3010275 | G/T | 0.208 | -0.019 | 0.002 | 1.55E-26 | 114 |
| LDL | rs4703645 | C/T | 0.164 | 0.061 | 0.002 | 1.00E-200 | 977 |
| LDL | rs6888261 | G/A | 0.071 | 0.035 | 0.003 | 4.49E-37 | 162 |
| LDL | rs72768389 | A/C | 0.019 | -0.045 | 0.006 | 1.87E-14 | 59 |
| LDL | rs62363367 | C/A | 0.02 | -0.028 | 0.005 | 4.93E-08 | 30 |
| LDL | rs2972831 | T/C | 0.112 | 0.017 | 0.002 | 1.41E-13 | 55 |
| LDL | rs2617447 | G/A | 0.429 | 0.008 | 0.001 | 8.70E-09 | 33 |
| LDL | rs6869845 | C/T | 0.449 | -0.016 | 0.001 | 1.43E-28 | 123 |
| LDL | rs11745587 | A/G | 0.353 | 0.016 | 0.001 | 6.28E-27 | 115 |
| LDL | rs548522421 | T/G | 0.293 | 0.01 | 0.002 | 4.85E-09 | 34 |
| LDL | rs13179861 | A/G | 0.176 | -0.012 | 0.002 | 3.60E-11 | 44 |
| LDL | rs11167778 | T/C | 0.106 | 0.022 | 0.002 | 4.95E-22 | 93 |
| LDL | rs543486395 | A/C | 0.024 | -0.048 | 0.006 | 1.60E-15 | 63 |
| LDL | rs12657266 | C/T | 0.366 | -0.035 | 0.001 | 4.18E-125 | 566 |
| LDL | rs13357800 | C/T | 0.43 | -0.01 | 0.001 | 1.75E-11 | 45 |
| LDL | rs352942 | A/G | 0.251 | -0.01 | 0.002 | 1.48E-09 | 37 |
| LDL | rs272441 | A/G | 0.091 | 0.014 | 0.003 | 1.44E-08 | 32 |
| LDL | rs9405990 | A/G | 0.499 | -0.008 | 0.001 | 3.10E-08 | 31 |
| LDL | rs59408219 | C/T | 0.079 | -0.022 | 0.003 | 3.44E-17 | 71 |
| LDL | rs2235215 | C/T | 0.319 | -0.031 | 0.002 | 2.19E-93 | 420 |
| LDL | rs6924805 | G/T | 0.41 | 0.009 | 0.001 | 3.70E-09 | 35 |
| LDL | rs9368188 | A/G | 0.334 | -0.009 | 0.002 | 6.83E-09 | 34 |
| LDL | rs113760175 | A/G | 0.064 | -0.02 | 0.003 | 1.95E-11 | 45 |
| LDL | rs3823151 | C/A | 0.031 | 0.023 | 0.004 | 8.58E-09 | 33 |
| LDL | rs1800562 | A/G | 0.07 | -0.057 | 0.003 | 6.06E-93 | 418 |
| LDL | rs148268586 | G/A | 0.016 | 0.036 | 0.006 | 2.32E-09 | 36 |
| LDL | rs71559014 | G/C | 0.078 | -0.032 | 0.003 | 5.24E-32 | 139 |
| LDL | rs35814746 | C/T | 0.081 | -0.027 | 0.003 | 3.23E-24 | 103 |
| LDL | rs62406559 | G/T | 0.033 | 0.026 | 0.004 | 2.52E-10 | 40 |
| LDL | rs2517671 | G/A | 0.407 | 0.015 | 0.002 | 4.79E-23 | 98 |
| LDL | rs1265097 | A/C | 0.092 | 0.029 | 0.002 | 2.22E-31 | 136 |
| LDL | rs2596544 | A/T | 0.201 | -0.011 | 0.002 | 9.28E-10 | 37 |
| LDL | rs28732146 | A/T | 0.197 | 0.015 | 0.002 | 1.74E-15 | 63 |
| LDL | rs28664894 | A/C | 0.213 | -0.015 | 0.003 | 5.52E-09 | 34 |
| LDL | rs6689 | G/A | 0.201 | 0.039 | 0.002 | 7.95E-89 | 399 |
| LDL | rs3800461 | C/G | 0.115 | -0.025 | 0.002 | 4.35E-29 | 125 |
| LDL | rs9470079 | A/G | 0.163 | -0.012 | 0.002 | 4.23E-10 | 39 |
| LDL | rs62406547 | C/A | 0.494 | -0.012 | 0.002 | 4.72E-15 | 61 |
| LDL | rs11759627 | T/C | 0.321 | 0.01 | 0.002 | 1.78E-10 | 41 |
| LDL | rs2395943 | A/G | 0.411 | 0.014 | 0.001 | 3.34E-21 | 89 |
| LDL | rs68137036 | G/A | 0.281 | -0.01 | 0.002 | 1.06E-09 | 37 |
| LDL | rs66629131 | G/C | 0.206 | 0.01 | 0.002 | 3.79E-09 | 35 |
| LDL | rs55959984 | G/A | 0.285 | -0.016 | 0.002 | 2.64E-23 | 99 |
| LDL | rs4715344 | T/C | 0.491 | -0.01 | 0.001 | 3.87E-13 | 53 |
| LDL | rs12662589 | C/G | 0.258 | 0.015 | 0.002 | 4.99E-21 | 89 |
| LDL | rs9496567 | A/G | 0.238 | -0.021 | 0.002 | 1.50E-35 | 155 |
| LDL | rs62419249 | A/G | 0.48 | 0.012 | 0.001 | 2.38E-17 | 72 |
| LDL | rs4946713 | A/C | 0.449 | -0.011 | 0.001 | 3.45E-15 | 62 |
| LDL | rs2798646 | C/A | 0.123 | -0.015 | 0.002 | 2.27E-11 | 45 |
| LDL | rs1556857 | C/T | 0.407 | -0.017 | 0.001 | 1.23E-31 | 137 |
| LDL | rs9388498 | T/G | 0.188 | -0.016 | 0.002 | 6.35E-16 | 65 |
| LDL | rs141783576 | C/G | 0.069 | 0.024 | 0.003 | 7.98E-14 | 56 |
| LDL | rs3890746 | C/T | 0.427 | -0.01 | 0.001 | 5.10E-12 | 48 |
| LDL | rs9399137 | C/T | 0.258 | -0.026 | 0.002 | 1.15E-57 | 256 |
| LDL | rs721795 | T/C | 0.018 | -0.037 | 0.005 | 3.55E-12 | 48 |
| LDL | rs72974722 | C/A | 0.176 | 0.021 | 0.002 | 2.05E-29 | 127 |
| LDL | rs679582 | G/A | 0.379 | 0.01 | 0.001 | 4.55E-11 | 43 |
| LDL | rs9480534 | G/A | 0.093 | -0.017 | 0.002 | 1.98E-12 | 49 |
| LDL | rs1871859 | T/C | 0.127 | 0.019 | 0.002 | 2.55E-18 | 76 |
| LDL | rs112170089 | A/G | 0.018 | 0.054 | 0.006 | 1.64E-18 | 77 |
| LDL | rs12208357 | T/C | 0.07 | 0.064 | 0.003 | 4.42E-118 | 534 |
| LDL | rs146534110 | T/G | 0.012 | 0.077 | 0.007 | 3.73E-30 | 130 |
| LDL | rs191765480 | C/G | 0.007 | -0.049 | 0.009 | 2.15E-08 | 31 |
| LDL | rs117733303 | G/A | 0.018 | 0.143 | 0.005 | 2.50E-156 | 710 |
| LDL | rs118039278 | A/G | 0.07 | 0.113 | 0.003 | 1.00E-200 | 1622 |
| LDL | rs117881880 | A/T | 0.013 | -0.043 | 0.006 | 2.32E-11 | 45 |
| LDL | rs12212146 | C/T | 0.069 | -0.025 | 0.003 | 3.11E-18 | 76 |
| LDL | rs61730955 | A/G | 0.056 | 0.018 | 0.003 | 1.15E-08 | 33 |
| LDL | rs10272002 | G/A | 0.208 | -0.022 | 0.002 | 9.17E-36 | 156 |
| LDL | rs28406917 | T/C | 0.423 | 0.013 | 0.001 | 3.73E-18 | 75 |
| LDL | rs55696093 | G/A | 0.211 | 0.038 | 0.002 | 3.61E-103 | 465 |
| LDL | rs4722551 | C/T | 0.16 | 0.035 | 0.002 | 1.04E-75 | 339 |
| LDL | rs6968895 | A/G | 0.105 | -0.014 | 0.002 | 1.25E-09 | 37 |
| LDL | rs4719925 | G/A | 0.091 | 0.014 | 0.002 | 1.80E-08 | 32 |
| LDL | rs12533280 | T/C | 0.193 | 0.018 | 0.002 | 2.59E-23 | 99 |
| LDL | rs7808613 | G/C | 0.244 | 0.011 | 0.002 | 1.72E-10 | 41 |
| LDL | rs710887 | T/C | 0.319 | -0.023 | 0.002 | 1.60E-45 | 201 |
| LDL | rs799157 | T/C | 0.037 | 0.033 | 0.004 | 7.03E-17 | 70 |
| LDL | rs2302434 | T/C | 0.167 | 0.014 | 0.002 | 1.65E-13 | 54 |
| LDL | rs4148826 | C/T | 0.182 | -0.013 | 0.002 | 4.98E-13 | 52 |
| LDL | rs6967728 | A/G | 0.184 | -0.018 | 0.002 | 1.05E-21 | 92 |
| LDL | rs221793 | A/C | 0.099 | 0.028 | 0.002 | 1.53E-31 | 137 |
| LDL | rs13233422 | T/C | 0.222 | -0.01 | 0.002 | 3.20E-09 | 35 |
| LDL | rs803073 | A/G | 0.476 | -0.008 | 0.001 | 2.06E-08 | 31 |
| LDL | rs10241374 | A/G | 0.119 | -0.012 | 0.002 | 4.35E-08 | 30 |
| LDL | rs10248717 | G/A | 0.31 | 0.013 | 0.002 | 4.56E-16 | 66 |
| LDL | rs62621812 | A/G | 0.023 | 0.027 | 0.005 | 2.02E-08 | 31 |
| LDL | rs1838931 | T/C | 0.312 | 0.012 | 0.002 | 1.66E-13 | 54 |
| LDL | rs34372369 | A/G | 0.05 | 0.02 | 0.003 | 1.44E-09 | 37 |
| LDL | rs4374942 | C/T | 0.077 | 0.02 | 0.003 | 5.33E-14 | 57 |
| LDL | rs2928576 | T/C | 0.329 | -0.012 | 0.002 | 3.34E-16 | 67 |
| LDL | rs330082 | G/C | 0.092 | 0.014 | 0.003 | 1.47E-08 | 32 |
| LDL | rs9987289 | A/G | 0.089 | -0.06 | 0.002 | 3.18E-128 | 580 |
| LDL | rs6601302 | T/G | 0.248 | 0.015 | 0.002 | 1.78E-19 | 81 |
| LDL | rs11250076 | A/G | 0.424 | 0.01 | 0.001 | 1.18E-12 | 51 |
| LDL | rs11993679 | C/T | 0.222 | -0.012 | 0.002 | 2.99E-12 | 49 |
| LDL | rs1495741 | G/A | 0.22 | 0.021 | 0.002 | 1.10E-35 | 155 |
| LDL | rs900776 | C/A | 0.167 | -0.019 | 0.002 | 5.77E-23 | 97 |
| LDL | rs7837764 | C/G | 0.381 | -0.008 | 0.001 | 2.65E-08 | 31 |
| LDL | rs6557915 | C/A | 0.286 | -0.009 | 0.002 | 1.79E-08 | 32 |
| LDL | rs117908407 | A/G | 0.034 | 0.03 | 0.004 | 9.84E-15 | 60 |
| LDL | rs117139027 | A/G | 0.014 | -0.077 | 0.006 | 6.62E-33 | 143 |
| LDL | rs34265667 | A/G | 0.032 | -0.025 | 0.004 | 3.24E-10 | 40 |
| LDL | rs9298506 | G/A | 0.207 | 0.024 | 0.002 | 7.47E-42 | 184 |
| LDL | rs9297994 | G/A | 0.336 | 0.035 | 0.002 | 2.83E-122 | 553 |
| LDL | rs12114596 | T/C | 0.345 | 0.012 | 0.002 | 4.89E-13 | 52 |
| LDL | rs62509311 | T/A | 0.279 | -0.015 | 0.002 | 5.41E-22 | 93 |
| LDL | rs2941465 | T/C | 0.432 | -0.009 | 0.001 | 2.29E-09 | 36 |
| LDL | rs400824 | T/C | 0.293 | 0.011 | 0.002 | 3.11E-11 | 44 |
| LDL | rs4464946 | A/G | 0.247 | -0.009 | 0.002 | 3.47E-08 | 30 |
| LDL | rs2737245 | T/G | 0.275 | -0.025 | 0.002 | 5.76E-56 | 248 |
| LDL | rs2385094 | C/T | 0.124 | -0.015 | 0.002 | 4.59E-12 | 48 |
| LDL | rs6999569 | G/A | 0.472 | -0.054 | 0.001 | 1.00E-200 | 1422 |
| LDL | rs45613837 | A/G | 0.447 | -0.008 | 0.001 | 4.53E-08 | 30 |
| LDL | rs1134030 | T/C | 0.363 | 0.022 | 0.002 | 1.13E-49 | 220 |
| LDL | rs3780181 | G/A | 0.07 | -0.035 | 0.003 | 1.17E-35 | 155 |
| LDL | rs28498684 | A/G | 0.399 | 0.012 | 0.001 | 5.79E-17 | 70 |
| LDL | rs12551960 | T/C | 0.08 | 0.035 | 0.003 | 1.47E-37 | 164 |
| LDL | rs10757273 | A/C | 0.447 | -0.014 | 0.002 | 1.70E-18 | 77 |
| LDL | rs139956529 | T/C | 0.017 | 0.034 | 0.006 | 1.78E-08 | 32 |
| LDL | rs7864568 | A/G | 0.309 | -0.016 | 0.002 | 1.91E-22 | 95 |
| LDL | rs1571791 | T/C | 0.381 | 0.014 | 0.001 | 9.65E-22 | 92 |
| LDL | rs9410207 | C/T | 0.067 | -0.019 | 0.003 | 1.36E-11 | 46 |
| LDL | rs2401637 | T/C | 0.336 | -0.009 | 0.002 | 1.24E-08 | 32 |
| LDL | rs2066714 | C/T | 0.125 | 0.019 | 0.002 | 1.90E-18 | 77 |
| LDL | rs11789603 | T/C | 0.102 | 0.023 | 0.002 | 2.13E-22 | 95 |
| LDL | rs2740488 | C/A | 0.264 | -0.025 | 0.002 | 5.50E-55 | 244 |
| LDL | rs56294298 | A/G | 0.086 | -0.018 | 0.003 | 3.94E-12 | 48 |
| LDL | rs13289095 | T/G | 0.139 | -0.029 | 0.002 | 1.68E-42 | 187 |
| LDL | rs2519093 | T/C | 0.187 | 0.072 | 0.002 | 1.00E-200 | 1590 |
| LDL | rs41307428 | T/C | 0.025 | -0.028 | 0.005 | 4.02E-10 | 39 |
| LDL | rs3780190 | A/G | 0.464 | 0.012 | 0.001 | 1.78E-15 | 63 |
| LDL | rs13301660 | T/C | 0.271 | -0.016 | 0.002 | 5.21E-22 | 93 |
| LDL | rs263423 | A/G | 0.202 | -0.011 | 0.002 | 7.12E-10 | 38 |
| LDL | rs7903259 | G/C | 0.416 | 0.016 | 0.001 | 3.57E-29 | 126 |
| LDL | rs12354765 | A/T | 0.249 | -0.01 | 0.002 | 1.85E-08 | 32 |
| LDL | rs7908745 | G/A | 0.311 | 0.009 | 0.002 | 4.38E-09 | 34 |
| LDL | rs10761750 | A/G | 0.484 | 0.014 | 0.001 | 1.34E-22 | 96 |
| LDL | rs17476364 | C/T | 0.101 | -0.027 | 0.002 | 1.45E-29 | 127 |
| LDL | rs11000443 | A/C | 0.044 | -0.023 | 0.003 | 1.47E-11 | 46 |
| LDL | rs1870140 | A/G | 0.155 | -0.013 | 0.002 | 4.42E-11 | 43 |
| LDL | rs477418 | A/C | 0.034 | -0.025 | 0.004 | 1.21E-10 | 41 |
| LDL | rs2250781 | C/A | 0.477 | 0.009 | 0.001 | 1.58E-09 | 36 |
| LDL | rs2068888 | A/G | 0.451 | -0.018 | 0.001 | 7.80E-35 | 152 |
| LDL | rs28371678 | C/T | 0.054 | -0.021 | 0.003 | 2.28E-10 | 40 |
| LDL | rs603424 | A/G | 0.171 | 0.016 | 0.002 | 9.59E-17 | 69 |
| LDL | rs76633616 | C/T | 0.062 | 0.019 | 0.003 | 1.59E-10 | 41 |
| LDL | rs2792751 | T/C | 0.279 | 0.023 | 0.002 | 1.65E-49 | 219 |
| LDL | rs17672352 | T/C | 0.181 | -0.018 | 0.002 | 2.59E-23 | 99 |
| LDL | rs72823014 | A/G | 0.12 | -0.016 | 0.002 | 1.85E-13 | 54 |
| LDL | rs2301179 | A/G | 0.496 | -0.016 | 0.001 | 7.35E-29 | 124 |
| LDL | rs7904973 | G/T | 0.426 | -0.021 | 0.001 | 1.52E-47 | 210 |
| LDL | rs11246279 | A/C | 0.474 | 0.008 | 0.001 | 1.73E-08 | 32 |
| LDL | rs151191319 | A/G | 0.031 | -0.028 | 0.004 | 4.91E-12 | 48 |
| LDL | rs1661052 | G/A | 0.092 | -0.016 | 0.003 | 1.71E-10 | 41 |
| LDL | rs7108486 | C/T | 0.025 | -0.031 | 0.005 | 3.05E-11 | 44 |
| LDL | rs12271333 | C/A | 0.135 | 0.016 | 0.002 | 1.44E-14 | 59 |
| LDL | rs11601507 | A/C | 0.069 | 0.041 | 0.003 | 1.37E-34 | 150 |
| LDL | rs7944706 | A/G | 0.424 | 0.009 | 0.002 | 5.31E-09 | 34 |
| LDL | rs11022130 | G/A | 0.25 | 0.01 | 0.002 | 8.47E-09 | 33 |
| LDL | rs11023881 | A/T | 0.398 | -0.008 | 0.001 | 2.10E-08 | 31 |
| LDL | rs214082 | T/C | 0.413 | -0.008 | 0.001 | 2.45E-08 | 31 |
| LDL | rs10832956 | T/C | 0.267 | -0.02 | 0.002 | 2.11E-36 | 159 |
| LDL | rs2349805 | C/G | 0.105 | 0.014 | 0.003 | 2.77E-08 | 31 |
| LDL | rs223042 | C/G | 0.136 | 0.012 | 0.002 | 1.01E-08 | 33 |
| LDL | rs61882722 | T/C | 0.031 | -0.026 | 0.004 | 3.77E-10 | 39 |
| LDL | rs1631174 | A/C | 0.341 | 0.01 | 0.002 | 2.86E-10 | 40 |
| LDL | rs11230285 | G/A | 0.31 | -0.009 | 0.002 | 2.74E-08 | 31 |
| LDL | rs174547 | C/T | 0.343 | -0.042 | 0.001 | 2.73E-171 | 778 |
| LDL | rs77631946 | A/C | 0.093 | -0.019 | 0.003 | 7.96E-14 | 56 |
| LDL | rs56236098 | C/T | 0.135 | 0.014 | 0.002 | 1.17E-10 | 42 |
| LDL | rs17495838 | C/T | 0.235 | -0.015 | 0.002 | 2.91E-19 | 80 |
| LDL | rs55987642 | T/C | 0.053 | -0.024 | 0.003 | 1.44E-13 | 55 |
| LDL | rs78643851 | T/G | 0.022 | 0.034 | 0.006 | 1.14E-09 | 37 |
| LDL | rs11237488 | T/C | 0.124 | -0.014 | 0.002 | 4.11E-10 | 39 |
| LDL | rs12295353 | C/T | 0.478 | -0.008 | 0.001 | 2.48E-08 | 31 |
| LDL | rs11226108 | C/G | 0.192 | -0.015 | 0.002 | 3.56E-15 | 62 |
| LDL | rs964184 | G/C | 0.134 | 0.055 | 0.002 | 4.77E-154 | 699 |
| LDL | rs141469619 | G/A | 0.009 | 0.073 | 0.008 | 1.85E-19 | 81 |
| LDL | rs12970 | A/G | 0.061 | -0.027 | 0.003 | 2.40E-19 | 81 |
| LDL | rs4639966 | C/T | 0.24 | 0.014 | 0.002 | 2.14E-17 | 72 |
| LDL | rs3862606 | G/A | 0.448 | 0.01 | 0.001 | 7.74E-12 | 47 |
| LDL | rs1945390 | C/T | 0.393 | 0.015 | 0.001 | 8.45E-26 | 110 |
| LDL | rs112771035 | G/C | 0.069 | 0.062 | 0.003 | 4.74E-108 | 488 |
| LDL | rs181897168 | A/G | 0.009 | -0.07 | 0.008 | 2.11E-16 | 67 |
| LDL | rs111299622 | C/T | 0.023 | -0.03 | 0.005 | 6.55E-10 | 38 |
| LDL | rs35882350 | G/A | 0.255 | 0.018 | 0.002 | 4.80E-27 | 116 |
| LDL | rs76895963 | G/T | 0.018 | -0.057 | 0.006 | 1.19E-22 | 96 |
| LDL | rs75667995 | C/T | 0.066 | -0.028 | 0.003 | 2.12E-22 | 95 |
| LDL | rs1007938 | G/A | 0.409 | 0.009 | 0.001 | 1.26E-09 | 37 |
| LDL | rs10843390 | T/C | 0.279 | -0.009 | 0.002 | 3.12E-09 | 35 |
| LDL | rs10784340 | T/C | 0.061 | 0.028 | 0.003 | 1.49E-20 | 86 |
| LDL | rs2250751 | A/G | 0.342 | -0.017 | 0.002 | 1.12E-29 | 128 |
| LDL | rs10876169 | A/T | 0.408 | -0.013 | 0.001 | 1.72E-19 | 82 |
| LDL | rs4919739 | G/T | 0.375 | -0.009 | 0.001 | 2.98E-09 | 35 |
| LDL | rs114075368 | G/T | 0.174 | 0.014 | 0.002 | 1.16E-13 | 55 |
| LDL | rs1078604 | T/G | 0.071 | -0.015 | 0.003 | 2.29E-08 | 31 |
| LDL | rs2122982 | A/G | 0.247 | -0.014 | 0.002 | 2.00E-17 | 72 |
| LDL | rs74093304 | A/G | 0.123 | 0.013 | 0.002 | 4.85E-09 | 34 |
| LDL | rs61754230 | T/C | 0.016 | 0.049 | 0.006 | 2.75E-17 | 72 |
| LDL | rs11105294 | A/G | 0.342 | 0.012 | 0.002 | 1.03E-14 | 60 |
| LDL | rs1515565 | A/G | 0.486 | -0.01 | 0.001 | 2.19E-11 | 45 |
| LDL | rs1095974 | G/C | 0.093 | -0.014 | 0.002 | 2.92E-08 | 31 |
| LDL | rs1536464 | C/T | 0.455 | -0.01 | 0.001 | 7.60E-13 | 51 |
| LDL | rs978458 | T/C | 0.26 | 0.012 | 0.002 | 4.96E-14 | 57 |
| LDL | rs1850956 | T/C | 0.5 | -0.008 | 0.001 | 2.06E-08 | 31 |
| LDL | rs1196760 | G/C | 0.094 | 0.021 | 0.002 | 3.81E-17 | 71 |
| LDL | rs11114033 | A/G | 0.355 | 0.012 | 0.001 | 7.54E-16 | 65 |
| LDL | rs118014788 | T/G | 0.029 | 0.028 | 0.004 | 3.66E-10 | 39 |
| LDL | rs653178 | C/T | 0.477 | -0.023 | 0.001 | 6.89E-60 | 266 |
| LDL | rs10850071 | C/G | 0.304 | -0.015 | 0.002 | 7.67E-22 | 92 |
| LDL | rs12811045 | G/A | 0.211 | 0.01 | 0.002 | 1.14E-08 | 33 |
| LDL | rs1169288 | C/A | 0.319 | 0.036 | 0.002 | 4.23E-122 | 552 |
| LDL | rs1168674 | C/T | 0.132 | 0.013 | 0.002 | 6.86E-10 | 38 |
| LDL | rs28461471 | G/A | 0.095 | 0.022 | 0.003 | 2.84E-16 | 67 |
| LDL | rs1716393 | G/T | 0.402 | -0.01 | 0.001 | 1.08E-12 | 51 |
| LDL | rs11057840 | C/A | 0.138 | 0.021 | 0.002 | 5.67E-25 | 107 |
| LDL | rs75588192 | A/G | 0.14 | 0.016 | 0.002 | 4.90E-13 | 52 |
| LDL | rs11611651 | A/G | 0.086 | 0.014 | 0.003 | 4.45E-08 | 30 |
| LDL | rs76428106 | C/T | 0.012 | -0.039 | 0.007 | 3.79E-08 | 30 |
| LDL | rs5026917 | C/A | 0.379 | -0.008 | 0.002 | 1.98E-08 | 32 |
| LDL | rs7327867 | G/A | 0.476 | 0.02 | 0.001 | 4.47E-44 | 194 |
| LDL | rs208432 | C/T | 0.386 | 0.01 | 0.001 | 1.31E-11 | 46 |
| LDL | rs17532371 | G/C | 0.072 | -0.019 | 0.003 | 2.01E-12 | 49 |
| LDL | rs7317982 | T/C | 0.47 | 0.009 | 0.001 | 5.15E-10 | 39 |
| LDL | rs201798 | G/A | 0.381 | 0.008 | 0.001 | 2.41E-08 | 31 |
| LDL | rs9535755 | G/C | 0.172 | 0.012 | 0.002 | 7.02E-10 | 38 |
| LDL | rs9592980 | G/A | 0.412 | -0.01 | 0.001 | 1.69E-11 | 45 |
| LDL | rs67896106 | A/C | 0.134 | 0.012 | 0.002 | 7.16E-09 | 33 |
| LDL | rs7336371 | A/G | 0.232 | 0.015 | 0.002 | 3.79E-19 | 80 |
| LDL | rs4771674 | A/G | 0.379 | -0.014 | 0.001 | 2.22E-22 | 95 |
| LDL | rs6602909 | C/T | 0.33 | 0.021 | 0.002 | 1.98E-42 | 186 |
| LDL | rs12016920 | C/T | 0.196 | -0.017 | 0.002 | 4.65E-21 | 89 |
| LDL | rs11621792 | T/C | 0.453 | 0.022 | 0.001 | 2.60E-53 | 236 |
| LDL | rs139262716 | A/G | 0.019 | -0.037 | 0.005 | 1.22E-11 | 46 |
| LDL | rs11846704 | T/C | 0.267 | -0.013 | 0.002 | 1.49E-16 | 68 |
| LDL | rs140022586 | G/T | 0.018 | 0.032 | 0.005 | 3.18E-09 | 35 |
| LDL | rs12897637 | C/T | 0.161 | 0.015 | 0.002 | 4.13E-15 | 62 |
| LDL | rs7155332 | C/G | 0.145 | -0.025 | 0.002 | 1.22E-35 | 155 |
| LDL | rs13379043 | C/T | 0.275 | -0.016 | 0.002 | 1.03E-24 | 105 |
| LDL | rs2058919 | G/C | 0.388 | 0.01 | 0.001 | 4.58E-11 | 43 |
| LDL | rs11622292 | C/T | 0.397 | 0.009 | 0.001 | 1.69E-10 | 41 |
| LDL | rs28929474 | T/C | 0.018 | 0.049 | 0.005 | 2.77E-20 | 85 |
| LDL | rs17580 | A/T | 0.04 | 0.05 | 0.004 | 1.25E-42 | 187 |
| LDL | rs8015761 | T/C | 0.455 | 0.008 | 0.001 | 4.41E-08 | 30 |
| LDL | rs2413926 | A/T | 0.376 | -0.013 | 0.001 | 2.14E-19 | 81 |
| LDL | rs17652767 | A/G | 0.103 | -0.013 | 0.002 | 4.38E-08 | 30 |
| LDL | rs79391862 | C/A | 0.02 | -0.065 | 0.006 | 3.94E-31 | 135 |
| LDL | rs72749499 | G/A | 0.072 | 0.029 | 0.003 | 2.33E-24 | 104 |
| LDL | rs4775039 | G/C | 0.458 | 0.009 | 0.002 | 1.22E-08 | 32 |
| LDL | rs261342 | G/C | 0.215 | 0.015 | 0.002 | 7.66E-17 | 69 |
| LDL | rs1836996 | C/T | 0.474 | -0.008 | 0.001 | 4.25E-08 | 30 |
| LDL | rs11636087 | C/T | 0.297 | 0.015 | 0.002 | 1.18E-20 | 87 |
| LDL | rs6495122 | A/C | 0.421 | -0.012 | 0.001 | 6.12E-17 | 70 |
| LDL | rs9920066 | A/T | 0.352 | -0.009 | 0.002 | 6.33E-10 | 38 |
| LDL | rs8029797 | A/T | 0.314 | -0.01 | 0.002 | 4.42E-10 | 39 |
| LDL | rs17703146 | C/T | 0.163 | 0.012 | 0.002 | 3.68E-09 | 35 |
| LDL | rs12445804 | A/G | 0.075 | 0.033 | 0.003 | 7.26E-32 | 138 |
| LDL | rs246184 | C/G | 0.327 | -0.009 | 0.002 | 2.34E-08 | 31 |
| LDL | rs72772042 | A/C | 0.148 | -0.012 | 0.002 | 4.04E-10 | 39 |
| LDL | rs35468353 | G/A | 0.377 | 0.012 | 0.001 | 3.97E-16 | 66 |
| LDL | rs247617 | A/C | 0.323 | -0.036 | 0.002 | 1.05E-124 | 564 |
| LDL | rs181501802 | A/G | 0.061 | 0.023 | 0.003 | 3.75E-13 | 53 |
| LDL | rs56212732 | T/C | 0.083 | -0.026 | 0.003 | 2.41E-19 | 81 |
| LDL | rs11075892 | C/T | 0.373 | 0.013 | 0.001 | 6.41E-18 | 74 |
| LDL | rs12924886 | T/A | 0.191 | 0.057 | 0.002 | 1.00E-200 | 992 |
| LDL | rs7202323 | G/T | 0.229 | -0.022 | 0.002 | 2.06E-39 | 173 |
| LDL | rs7404072 | C/T | 0.286 | -0.011 | 0.002 | 3.64E-11 | 44 |
| LDL | rs1121985 | A/C | 0.372 | -0.01 | 0.001 | 5.37E-12 | 48 |
| LDL | rs1862719 | A/G | 0.247 | 0.011 | 0.002 | 9.07E-11 | 42 |
| LDL | rs78755089 | T/G | 0.08 | 0.016 | 0.003 | 8.07E-10 | 38 |
| LDL | rs67890964 | C/T | 0.374 | -0.018 | 0.002 | 1.53E-33 | 146 |
| LDL | rs13337963 | T/C | 0.346 | 0.01 | 0.002 | 8.24E-09 | 33 |
| LDL | rs56292801 | A/G | 0.266 | -0.013 | 0.002 | 2.03E-15 | 63 |
| LDL | rs140194134 | T/G | 0.052 | 0.033 | 0.004 | 1.32E-16 | 68 |
| LDL | rs10852932 | T/G | 0.358 | -0.009 | 0.001 | 2.29E-09 | 36 |
| LDL | rs8078118 | A/C | 0.414 | 0.014 | 0.001 | 4.42E-21 | 89 |
| LDL | rs200099887 | T/C | 0.024 | -0.028 | 0.005 | 2.54E-08 | 31 |
| LDL | rs55714927 | T/C | 0.185 | -0.035 | 0.002 | 2.84E-75 | 337 |
| LDL | rs150688657 | A/G | 0.104 | 0.022 | 0.002 | 2.28E-20 | 86 |
| LDL | rs2270445 | G/A | 0.483 | 0.014 | 0.001 | 3.99E-22 | 94 |
| LDL | rs17681684 | A/G | 0.32 | 0.01 | 0.002 | 6.21E-11 | 43 |
| LDL | rs28811342 | C/T | 0.199 | 0.013 | 0.002 | 1.55E-13 | 55 |
| LDL | rs9909417 | A/G | 0.281 | 0.009 | 0.002 | 8.66E-09 | 33 |
| LDL | rs704 | A/G | 0.48 | 0.018 | 0.001 | 7.20E-38 | 165 |
| LDL | rs8070966 | T/C | 0.155 | -0.015 | 0.002 | 2.10E-14 | 58 |
| LDL | rs117494024 | C/T | 0.084 | -0.02 | 0.003 | 1.09E-12 | 51 |
| LDL | rs146932505 | G/T | 0.018 | 0.034 | 0.006 | 6.62E-09 | 34 |
| LDL | rs12945088 | G/A | 0.305 | -0.017 | 0.002 | 1.79E-25 | 109 |
| LDL | rs533075 | C/T | 0.341 | 0.01 | 0.002 | 1.09E-10 | 42 |
| LDL | rs12943633 | T/C | 0.081 | -0.017 | 0.003 | 2.66E-10 | 40 |
| LDL | rs2354155 | G/A | 0.473 | 0.011 | 0.002 | 3.70E-12 | 48 |
| LDL | rs72836561 | T/C | 0.031 | -0.03 | 0.004 | 4.07E-13 | 53 |
| LDL | rs8072100 | A/T | 0.492 | 0.026 | 0.001 | 6.97E-73 | 326 |
| LDL | rs11079830 | G/A | 0.418 | -0.013 | 0.001 | 4.59E-19 | 80 |
| LDL | rs62078383 | C/A | 0.412 | 0.009 | 0.001 | 1.25E-09 | 37 |
| LDL | rs2150879 | G/A | 0.452 | -0.01 | 0.001 | 9.86E-12 | 46 |
| LDL | rs2854153 | T/C | 0.273 | -0.01 | 0.002 | 3.12E-09 | 35 |
| LDL | rs1801689 | C/A | 0.027 | 0.091 | 0.004 | 6.24E-99 | 446 |
| LDL | rs7212076 | C/G | 0.485 | 0.012 | 0.001 | 8.91E-18 | 74 |
| LDL | rs7210027 | C/T | 0.211 | 0.016 | 0.002 | 3.72E-20 | 85 |
| LDL | rs34931250 | T/C | 0.06 | 0.032 | 0.003 | 7.75E-26 | 110 |
| LDL | rs77542162 | G/A | 0.02 | 0.183 | 0.005 | 1.00E-200 | 1273 |
| LDL | rs72631343 | G/C | 0.129 | -0.045 | 0.002 | 6.41E-98 | 441 |
| LDL | rs4485425 | A/G | 0.289 | -0.018 | 0.002 | 7.37E-29 | 124 |
| LDL | rs2376583 | T/A | 0.499 | 0.017 | 0.001 | 1.46E-33 | 146 |
| LDL | rs7501528 | G/C | 0.198 | -0.012 | 0.002 | 1.48E-09 | 37 |
| LDL | rs11656152 | T/C | 0.179 | 0.012 | 0.002 | 5.58E-10 | 38 |
| LDL | rs2840354 | T/C | 0.193 | 0.012 | 0.002 | 1.88E-10 | 41 |
| LDL | rs1010810 | A/G | 0.116 | -0.013 | 0.002 | 5.47E-09 | 34 |
| LDL | rs76537328 | G/C | 0.13 | -0.012 | 0.002 | 1.17E-08 | 33 |
| LDL | rs77960347 | G/A | 0.013 | 0.07 | 0.006 | 1.03E-28 | 124 |
| LDL | rs10438978 | T/C | 0.18 | -0.018 | 0.002 | 3.35E-22 | 94 |
| LDL | rs12968116 | T/C | 0.126 | 0.014 | 0.002 | 8.13E-11 | 42 |
| LDL | rs402348 | G/T | 0.206 | -0.011 | 0.002 | 3.98E-10 | 39 |
| LDL | rs12454507 | G/A | 0.287 | 0.01 | 0.002 | 4.27E-09 | 34 |
| LDL | rs1135908 | T/G | 0.141 | 0.012 | 0.002 | 2.71E-09 | 35 |
| LDL | rs4807570 | A/G | 0.208 | -0.012 | 0.002 | 5.63E-12 | 47 |
| LDL | rs4807332 | C/T | 0.307 | 0.016 | 0.002 | 3.79E-23 | 98 |
| LDL | rs34054295 | A/G | 0.383 | 0.012 | 0.001 | 8.49E-16 | 65 |
| LDL | rs708686 | T/C | 0.276 | 0.011 | 0.002 | 1.94E-11 | 45 |
| LDL | rs571497 | A/G | 0.154 | -0.014 | 0.002 | 3.76E-13 | 53 |
| LDL | rs45518133 | T/C | 0.012 | -0.118 | 0.007 | 1.38E-73 | 329 |
| LDL | rs8110479 | T/C | 0.042 | -0.122 | 0.004 | 1.00E-200 | 1056 |
| LDL | rs141015559 | A/G | 0.014 | 0.048 | 0.007 | 6.23E-13 | 52 |
| LDL | rs77935048 | A/G | 0.009 | -0.049 | 0.008 | 1.27E-09 | 37 |
| LDL | rs78688793 | A/G | 0.017 | -0.047 | 0.006 | 2.94E-15 | 62 |
| LDL | rs34795914 | T/G | 0.028 | 0.061 | 0.005 | 7.63E-38 | 165 |
| LDL | rs116945003 | A/G | 0.007 | 0.081 | 0.009 | 4.99E-20 | 84 |
| LDL | rs73013176 | C/T | 0.012 | -0.217 | 0.007 | 1.00E-200 | 1009 |
| LDL | rs11668058 | A/G | 0.04 | 0.046 | 0.004 | 2.81E-36 | 158 |
| LDL | rs112159161 | T/C | 0.015 | -0.2 | 0.006 | 1.00E-200 | 1108 |
| LDL | rs116959285 | G/C | 0.041 | 0.096 | 0.004 | 1.60E-132 | 600 |
| LDL | rs117955899 | T/C | 0.012 | -0.058 | 0.007 | 5.71E-18 | 75 |
| LDL | rs147540853 | A/G | 0.026 | -0.086 | 0.004 | 2.11E-85 | 384 |
| LDL | rs56048141 | T/C | 0.017 | -0.06 | 0.006 | 5.89E-25 | 106 |
| LDL | rs4143973 | C/G | 0.488 | -0.009 | 0.001 | 5.66E-10 | 38 |
| LDL | rs10415758 | T/A | 0.417 | -0.01 | 0.002 | 1.96E-11 | 45 |
| LDL | rs660247 | A/G | 0.471 | -0.009 | 0.001 | 1.55E-10 | 41 |
| LDL | rs11881955 | G/A | 0.386 | 0.009 | 0.002 | 3.09E-08 | 31 |
| LDL | rs3745185 | A/G | 0.446 | -0.01 | 0.001 | 4.53E-11 | 43 |
| LDL | rs4808766 | C/G | 0.258 | 0.014 | 0.002 | 6.75E-18 | 74 |
| LDL | rs111436413 | T/C | 0.023 | -0.033 | 0.005 | 4.38E-10 | 39 |
| LDL | rs2228603 | T/C | 0.076 | -0.087 | 0.003 | 1.00E-200 | 1046 |
| LDL | rs187429064 | G/A | 0.01 | -0.163 | 0.008 | 1.15E-92 | 417 |
| LDL | rs34858588 | G/C | 0.079 | 0.018 | 0.003 | 1.99E-11 | 45 |
| LDL | rs140588027 | A/T | 0.024 | -0.054 | 0.005 | 4.58E-26 | 112 |
| LDL | rs147791730 | A/G | 0.028 | -0.03 | 0.004 | 7.24E-12 | 47 |
| LDL | rs916694 | T/C | 0.422 | -0.01 | 0.001 | 1.12E-11 | 46 |
| LDL | rs11881404 | A/G | 0.374 | 0.009 | 0.001 | 5.02E-09 | 34 |
| LDL | rs4803525 | A/G | 0.417 | 0.009 | 0.001 | 1.04E-10 | 42 |
| LDL | rs56113850 | T/C | 0.432 | -0.014 | 0.002 | 2.16E-20 | 86 |
| LDL | rs3213263 | T/C | 0.421 | -0.008 | 0.001 | 1.54E-08 | 32 |
| LDL | rs74747585 | C/T | 0.021 | -0.109 | 0.005 | 1.95E-100 | 453 |
| LDL | rs62119261 | C/A | 0.043 | -0.112 | 0.004 | 1.00E-200 | 986 |
| LDL | rs73048351 | A/C | 0.01 | -0.25 | 0.008 | 1.00E-200 | 1097 |
| LDL | rs4803748 | T/C | 0.386 | -0.076 | 0.002 | 1.00E-200 | 2482 |
| LDL | rs147711004 | A/G | 0.037 | 0.153 | 0.004 | 1.00E-200 | 1452 |
| LDL | rs141739979 | T/G | 0.009 | -0.05 | 0.008 | 5.44E-10 | 39 |
| LDL | rs117843462 | C/G | 0.016 | 0.053 | 0.006 | 1.82E-17 | 72 |
| LDL | rs113139066 | G/T | 0.009 | 0.094 | 0.009 | 8.64E-28 | 119 |
| LDL | rs8108762 | A/G | 0.319 | -0.029 | 0.002 | 6.35E-78 | 349 |
| LDL | rs34212898 | T/C | 0.043 | -0.05 | 0.003 | 1.16E-46 | 206 |
| LDL | rs62111293 | C/T | 0.146 | 0.013 | 0.002 | 1.38E-08 | 32 |
| LDL | rs8106592 | C/G | 0.301 | 0.011 | 0.002 | 1.51E-09 | 37 |
| LDL | rs1644350 | A/T | 0.131 | 0.015 | 0.002 | 4.56E-13 | 52 |
| LDL | rs681343 | T/C | 0.5 | 0.03 | 0.001 | 3.07E-100 | 452 |
| LDL | rs142385484 | T/C | 0.153 | -0.019 | 0.002 | 7.11E-20 | 83 |
| LDL | rs11668882 | C/T | 0.431 | 0.008 | 0.001 | 1.01E-08 | 33 |
| LDL | rs1029709 | T/C | 0.278 | 0.013 | 0.002 | 1.78E-15 | 63 |
| LDL | rs35313547 | C/T | 0.157 | -0.015 | 0.002 | 4.05E-14 | 57 |
| LDL | rs35081008 | T/C | 0.156 | -0.03 | 0.002 | 3.84E-53 | 235 |
| LDL | rs55716128 | T/C | 0.179 | 0.017 | 0.002 | 1.16E-19 | 82 |
| LDL | rs6139104 | T/A | 0.089 | -0.016 | 0.003 | 5.41E-10 | 39 |
| LDL | rs8958 | T/C | 0.391 | 0.008 | 0.001 | 2.14E-08 | 31 |
| LDL | rs73078112 | T/C | 0.025 | 0.046 | 0.005 | 4.58E-23 | 98 |
| LDL | rs62204032 | T/A | 0.049 | -0.021 | 0.004 | 5.54E-09 | 34 |
| LDL | rs364585 | A/G | 0.395 | -0.013 | 0.001 | 2.24E-20 | 86 |
| LDL | rs969075 | T/C | 0.338 | -0.015 | 0.002 | 3.07E-23 | 99 |
| LDL | rs2618566 | G/T | 0.339 | 0.04 | 0.002 | 8.08E-148 | 670 |
| LDL | rs1044573 | G/A | 0.494 | 0.01 | 0.001 | 1.15E-11 | 46 |
| LDL | rs7261820 | A/G | 0.139 | -0.031 | 0.002 | 1.00E-51 | 229 |
| LDL | rs73118985 | C/T | 0.035 | -0.028 | 0.004 | 1.64E-12 | 50 |
| LDL | rs1883711 | C/G | 0.031 | 0.129 | 0.004 | 2.62E-190 | 866 |
| LDL | rs35570186 | A/G | 0.037 | 0.027 | 0.004 | 3.77E-11 | 44 |
| LDL | rs6093446 | A/G | 0.276 | 0.024 | 0.002 | 1.13E-51 | 229 |
| LDL | rs3092194 | A/G | 0.211 | -0.013 | 0.002 | 4.69E-12 | 48 |
| LDL | rs11700304 | C/T | 0.381 | 0.01 | 0.001 | 1.61E-10 | 41 |
| LDL | rs1800961 | T/C | 0.033 | -0.055 | 0.004 | 1.22E-43 | 192 |
| LDL | rs3843763 | T/C | 0.266 | -0.012 | 0.002 | 5.66E-13 | 52 |
| LDL | rs2295027 | A/G | 0.325 | 0.012 | 0.002 | 5.12E-14 | 57 |
| LDL | rs6063965 | A/G | 0.11 | 0.013 | 0.002 | 1.61E-08 | 32 |
| LDL | rs6022851 | T/C | 0.446 | 0.012 | 0.001 | 1.12E-15 | 64 |
| LDL | rs3746778 | A/G | 0.407 | -0.013 | 0.002 | 1.92E-15 | 63 |
| LDL | rs2738759 | A/G | 0.077 | -0.016 | 0.003 | 1.22E-08 | 32 |
| LDL | rs8126001 | T/C | 0.484 | -0.014 | 0.002 | 6.61E-21 | 88 |
| LDL | rs73147887 | G/C | 0.216 | 0.016 | 0.002 | 4.99E-19 | 79 |
| LDL | rs12106385 | A/T | 0.019 | -0.037 | 0.006 | 4.03E-11 | 44 |
| LDL | rs2833487 | G/A | 0.048 | 0.032 | 0.003 | 1.61E-21 | 91 |
| LDL | rs11911615 | G/T | 0.323 | -0.009 | 0.002 | 2.09E-08 | 31 |
| LDL | rs1963676 | C/T | 0.429 | -0.014 | 0.001 | 3.24E-22 | 94 |
| LDL | rs5746498 | C/T | 0.239 | 0.014 | 0.002 | 7.92E-15 | 60 |
| LDL | rs165722 | C/T | 0.472 | -0.009 | 0.001 | 9.31E-11 | 42 |
| LDL | rs878825 | C/T | 0.199 | -0.013 | 0.002 | 1.90E-13 | 54 |
| LDL | rs5752963 | A/G | 0.036 | 0.027 | 0.004 | 1.33E-12 | 50 |
| LDL | rs117147052 | C/T | 0.053 | 0.02 | 0.003 | 2.12E-09 | 36 |
| LDL | rs5755688 | A/G | 0.358 | 0.013 | 0.001 | 4.19E-18 | 75 |
| LDL | rs147428040 | A/G | 0.045 | 0.021 | 0.004 | 1.69E-08 | 32 |
| LDL | rs4263216 | C/G | 0.322 | -0.01 | 0.002 | 1.32E-09 | 37 |
| LDL | rs760719 | T/C | 0.429 | 0.01 | 0.001 | 4.66E-11 | 43 |
| LDL | rs138335 | C/G | 0.344 | 0.014 | 0.002 | 3.32E-20 | 85 |
| LDL | rs3747207 | A/G | 0.222 | -0.014 | 0.002 | 6.93E-16 | 65 |
| LDL | rs4823460 | C/T | 0.391 | -0.01 | 0.001 | 3.57E-11 | 44 |
| LDL | rs13268 | G/A | 0.024 | -0.036 | 0.005 | 3.95E-14 | 57 |
| LDL | rs41329344 | T/C | 0.108 | 0.013 | 0.002 | 1.27E-08 | 32 |
| LDL | rs9616822 | A/G | 0.355 | 0.011 | 0.001 | 2.27E-13 | 54 |
| LDL | rs142550943 | A/G | 0.073 | 0.017 | 0.003 | 5.46E-09 | 34 |
