# Supplementary material for: Lipid-lowering drugs and essential hemorrhagic thrombocythemia’s risk: A drug-target Mendelian randomization study
Source: Medicine (Baltimore). 2026 May 29;105(22):e49077. doi: 10.1097/MD.0000000000049077 (PMC13225517; doi:10.1097/MD.0000000000049077)
Supplement: Supplementary file 3 [file medi-105-e49077-s003.docx]

| **Table S2.Associations between genetically proxied exposures (drug targets and LDL) and outcomes (Thrombocytaemia using FinnGen and control outcomes** | | | | | | | | |
| --- | --- | --- | --- | --- | --- | --- | --- | --- |
|  |  |  |  |  |  |  |  |  |
| Outcome | Exposure | Method | Nsnp | OR | Lower 95%CI | Upper 95%CI | pval | adjustP |
| Thrombocytaemia（FinnGen) | PCSK9 | Inverse variance weighted (multiplicative random effects) | 33 | 1.87 | 1.2 | 2.91 | 0.01 | 0.02 |
| Thrombocytaemia（FinnGen) | PCSK9 | MR Egger | 33 | 1.31 | 0.75 | 2.28 | 0.35 | NA |
| Thrombocytaemia（FinnGen) | PCSK9 | Weighted median | 33 | 1.39 | 0.88 | 2.21 | 0.16 | NA |
| Thrombocytaemia（FinnGen) | PCSK9 | Weighted mode | 33 | 1.69 | 1.04 | 2.73 | 0.04 | NA |
| Thrombocytaemia（FinnGen) | HMGCR | Inverse variance weighted (multiplicative random effects) | 20 | 0.76 | 0.34 | 1.69 | 0.51 | 0.51 |
| Thrombocytaemia（FinnGen) | HMGCR | MR Egger | 20 | 0.42 | 0.01 | 24.81 | 0.68 | NA |
| Thrombocytaemia（FinnGen) | HMGCR | Weighted median | 20 | 0.48 | 0.15 | 1.59 | 0.23 | NA |
| Thrombocytaemia（FinnGen) | HMGCR | Weighted mode | 20 | 0.45 | 0.15 | 1.36 | 0.17 | NA |
| Thrombocytaemia（FinnGen) | NPC1L1 | Inverse variance weighted (multiplicative random effects) | 10 | 0.19 | 0.05 | 0.73 | 0.02 | 0.03 |
| Thrombocytaemia（FinnGen) | NPC1L1 | MR Egger | 10 | 4.29E-03 | 3.67E-05 | 0.5 | 0.06 | NA |
| Thrombocytaemia（FinnGen) | NPC1L1 | Weighted median | 10 | 0.13 | 0.02 | 0.81 | 0.03 | NA |
| Thrombocytaemia（FinnGen) | NPC1L1 | Weighted mode | 10 | 0.09 | 0.01 | 0.55 | 0.03 | NA |
| Thrombocytaemia（FinnGen) | LDL | Inverse variance weighted (multiplicative random effects) | 551 | 1.08 | 0.87 | 1.34 | 0.5 | 0.67 |
| Thrombocytaemia（FinnGen) | LDL | MR Egger | 551 | 1.11 | 0.79 | 1.55 | 0.54 | NA |
| Thrombocytaemia（FinnGen) | LDL | Weighted median | 551 | 1.21 | 0.86 | 1.69 | 0.27 | NA |
| Thrombocytaemia（FinnGen) | LDL | Weighted mode | 551 | 1.19 | 0.81 | 1.73 | 0.37 | NA |
| CAD（GWAS Catalog） | PCSK9 | Inverse variance weighted (multiplicative random effects) | 27 | 1.97 | 1.77 | 2.2 | 5.38E-35 | 1.08E-34 |
| CAD（GWAS Catalog） | PCSK9 | MR Egger | 27 | 2 | 1.62 | 2.47 | 9.05E-07 | NA |
| CAD（GWAS Catalog） | PCSK9 | Weighted median | 27 | 2.05 | 1.72 | 2.44 | 1.05E-15 | NA |
| CAD（GWAS Catalog） | PCSK9 | Weighted mode | 27 | 1.98 | 1.66 | 2.36 | 3.65E-08 | NA |
| CAD（GWAS Catalog） | HMGCR | Inverse variance weighted (multiplicative random effects) | 15 | 1.64 | 1.27 | 2.11 | 1.51E-04 | 1.51E-04 |
| CAD（GWAS Catalog） | HMGCR | MR Egger | 15 | 1.83 | 0.55 | 6.08 | 0.35 | NA |
| CAD（GWAS Catalog） | HMGCR | Weighted median | 15 | 1.44 | 0.99 | 2.08 | 0.05 | NA |
| CAD（GWAS Catalog） | HMGCR | Weighted mode | 15 | 1.4 | 0.97 | 2.03 | 0.09 | NA |
| CAD（GWAS Catalog） | NPC1L1 | Inverse variance weighted (multiplicative random effects) | 9 | 2.11 | 1.48 | 3.01 | 3.54E-05 | 4.72E-05 |
| CAD（GWAS Catalog） | NPC1L1 | MR Egger | 9 | 0.87 | 0.21 | 3.54 | 0.85 | NA |
| CAD（GWAS Catalog） | NPC1L1 | Weighted median | 9 | 1.81 | 1.13 | 2.92 | 0.01 | NA |
| CAD（GWAS Catalog） | NPC1L1 | Weighted mode | 9 | 1.76 | 1.13 | 2.76 | 0.04 | NA |
| CAD（GWAS Catalog） | LDL | Inverse variance weighted (multiplicative random effects) | 452 | 1.88 | 1.79 | 1.98 | 9.37E-129 | 3.75E-128 |
| CAD（GWAS Catalog） | LDL | MR Egger | 452 | 1.93 | 1.76 | 2.11 | 5.53E-38 | NA |
| CAD（GWAS Catalog） | LDL | Weighted median | 452 | 1.87 | 1.7 | 2.05 | 2.21E-37 | NA |
| CAD（GWAS Catalog） | LDL | Weighted mode | 452 | 1.88 | 1.66 | 2.12 | 3.16E-22 | NA |
