# Supplementary material for: Lipid-lowering drugs and essential hemorrhagic thrombocythemia’s risk: A drug-target Mendelian randomization study
Source: Medicine (Baltimore). 2026 May 29;105(22):e49077. doi: 10.1097/MD.0000000000049077 (PMC13225517; doi:10.1097/MD.0000000000049077)
Supplement: Supplementary file 4 [file medi-105-e49077-s004.docx]

| **Table S3. MR Egger intercepts.** | | | |
| --- | --- | --- | --- |
|  |  |  |  |
| **Outcome** | **Exposure** | **Egger intercept** | **p-val** |
| Thrombocytaemia（FinnGen) | PCSK9 | -0.04 | 0.06 |
| Thrombocytaemia（FinnGen) | NPC1L1 | -0.13 | 0.14 |
| Thrombocytaemia（FinnGen) | HMGCR | -0.03 | 0.77 |
| Thrombocytaemia（FinnGen) | LDL | 9.00E-04 | 0.82 |
| CAD（GWAS Catalog） | PCSK9 | -1.04E-03 | 0.84 |
| CAD（GWAS Catalog） | NPC1L1 | 0.03 | 0.24 |
| CAD（GWAS Catalog） | HMGCR | -5.20E-03 | 0.86 |
| CAD（GWAS Catalog） | LDL | -6.77E-04 | 0.48 |
