# Supplementary material for: Lipid-lowering drugs and essential hemorrhagic thrombocythemia’s risk: A drug-target Mendelian randomization study
Source: Medicine (Baltimore). 2026 May 29;105(22):e49077. doi: 10.1097/MD.0000000000049077 (PMC13225517; doi:10.1097/MD.0000000000049077)
Supplement: Supplementary file 5 [file medi-105-e49077-s005.docx]

Table S4. Colocalization results(PCSK9)

| PP.H0 | PP.H1 | PP.H2 | PP.H3 | PP.H4 | PP.H4/PP.H3+PP.H4 |
| --- | --- | --- | --- | --- | --- |
| 0.00E+00 | 9.11E-01 | 0.00E+00 | 1.37E-02 | 7.54E-02 | 8.46E-01 |
| Posterior probability for  PP.H0: neither trait has a genetic association in the region;  PP.H1: only trait 1 has a genetic association in the region;  PP.H2: only trait 2 has a genetic association in the region;  PP.H3: both traits are associated, but with different causal variants;  PP.H4: both traits are associated and share a single causal variant.  PP.H4/(PP.H3+PP.H4) represents the probability of colocalization conditional on the  presence of a causal variant for the outcome | | | | | |

Colocalization results(NPC1L1)

| PP.H0 | PP.H1 | PP.H2 | PP.H3 | PP.H4 | PP.H4/PP.H3+PP.H4 |
| --- | --- | --- | --- | --- | --- |
| 9.80e-01 | 3.98e-03 | 1.55e-02 | 6.28e-05 | 2.66e-04 | 8.09E-01 |
| Posterior probability for  PP.H0: neither trait has a genetic association in the region;  PP.H1: only trait 1 has a genetic association in the region;  PP.H2: only trait 2 has a genetic association in the region;  PP.H3: both traits are associated, but with different causal variants;  PP.H4: both traits are associated and share a single causal variant.  PP.H4/(PP.H3+PP.H4) represents the probability of colocalization conditional on the  presence of a causal variant for the outcome | | | | | |
